# Functional interpretation of ATAD3A variants in neuro-mitochondrial phenotypes

**DOI:** 10.1186/s13073-021-00873-3

**Published:** 2021-04-12

**Authors:** Zheng Yie Yap, Yo Han Park, Saskia B. Wortmann, Adam C. Gunning, Shlomit Ezer, Sukyeong Lee, Lita Duraine, Ekkehard Wilichowski, Kate Wilson, Johannes A. Mayr, Matias Wagner, Hong Li, Usha Kini, Emily Davis Black, Kristin G. Monaghan, James R. Lupski, Sian Ellard, Dominik S. Westphal, Tamar Harel, Wan Hee Yoon

**Affiliations:** 1 Aging and Metabolism Research Program, Oklahoma Medical Research Foundation, Oklahoma City, OK 73104 USA; 2 Institute of Human Genetics, Technical University Munich, Munich, Germany; 3 University Children’s Hospital, Paracelsus Medical University (PMU), Salzburg, Austria; 4 Radboud Centre for Mitochondrial Medicine (RCMM), Amalia Children’s Hospital, Nijmegen, The Netherlands; 5 Exeter Genomics Laboratory, Royal Devon and Exeter NHS Foundation Trust, Exeter, EX2 5DW UK; 6 Institute of Biomedical and Clinical Science, College of Medicine and Health, University of Exeter, Exeter, EX2 5DW UK; 7 Department of Genetics, Hadassah Medical Center, POB 12000, 9112001 Jerusalem, Israel; 8 Faculty of Medicine, Hebrew University of Jerusalem, POB 12000, 9112001 Jerusalem, Israel; 9 Verna and Marrs McLean Department of Biochemistry and Molecular Biology, Baylor College of Medicine, Houston, TX USA; 10 Jan and Dan Duncan Neurological Research Institute, Baylor College of Medicine, Houston, TX USA; 11 Department of Pediatrics and Pediatric Neurology, University Medical Center Göttingen, Georg-August-Universität Göttingen, Göttingen, Germany; 12 Oxford Centre for Genomic Medicine, Oxford University Hospitals NHS Foundation Trust, Oxford, UK; 13 Institute of Neurogenomics, Helmholtz Zentrum München, Neuherberg, Germany; 14 Department of Human Genetics, School of Medicine, Emory University, Atlanta, GA USA; 15 Department of Pediatrics, School of Medicine, Children’s Healthcare of Atlanta, Emory University, Atlanta, GA USA; 16 GeneDx Inc., Gaithersburg, MD USA; 17 Department of Molecular and Human Genetics, Baylor College of Medicine, Houston, TX USA; 18 Department of Pediatrics, Baylor College of Medicine, Houston, TX USA; 19 Human Genome Sequencing Center, Baylor College of Medicine, Houston, TX USA; 20 Texas Children’s Hospital, Houston, TX USA

**Keywords:** ATAD3A, Mitochondria, Disease, Autosomal recessive, Autophagy, Neurogenesis, *Drosophila*, AAA+ protein

## Abstract

**Background:**

ATPase family AAA-domain containing protein 3A (ATAD3A) is a nuclear-encoded mitochondrial membrane-anchored protein involved in diverse processes including mitochondrial dynamics, mitochondrial DNA organization, and cholesterol metabolism. Biallelic deletions (null), recessive missense variants (hypomorph), and heterozygous missense variants or duplications (antimorph) in *ATAD3A* lead to neurological syndromes in humans.

**Methods:**

To expand the mutational spectrum of *ATAD3A* variants and to provide functional interpretation of missense alleles in trans to deletion alleles, we performed exome sequencing for identification of single nucleotide variants (SNVs) and copy number variants (CNVs) in *ATAD3A* in individuals with neurological and mitochondrial phenotypes. A *Drosophila Atad3a Gal4* knockin-null allele was generated using CRISPR-Cas9 genome editing technology to aid the interpretation of variants.

**Results:**

We report 13 individuals from 8 unrelated families with biallelic *ATAD3A* variants. The variants included four missense variants inherited in trans to loss-of-function alleles (p.(Leu77Val), p.(Phe50Leu), p.(Arg170Trp), p.(Gly236Val)), a homozygous missense variant p.(Arg327Pro), and a heterozygous non-frameshift indel p.(Lys568del). Affected individuals exhibited findings previously associated with *ATAD3A* pathogenic variation, including developmental delay, hypotonia, congenital cataracts, hypertrophic cardiomyopathy, and cerebellar atrophy. *Drosophila* studies indicated that Phe50Leu, Gly236Val, Arg327Pro, and Lys568del are severe loss-of-function alleles leading to early developmental lethality. Further, we showed that Phe50Leu, Gly236Val, and Arg327Pro cause neurogenesis defects. On the contrary, Leu77Val and Arg170Trp are partial loss-of-function alleles that cause progressive locomotion defects and whose expression leads to an increase in autophagy and mitophagy in adult muscles.

**Conclusion:**

Our findings expand the allelic spectrum of *ATAD3A* variants and exemplify the use of a functional assay in *Drosophila* to aid variant interpretation.

## Background

The ATPase family AAA-domain containing protein 3A (ATAD3A) belongs to the ATPases associated with diverse cellular activities (AAA+ ATPase) protein family. It was initially identified as a mitochondrial protein enriched at contact sites between the mitochondria and the endoplasmic reticulum (ER) membrane [1]. The protein is presumed to tether the inner mitochondrial membrane to the outer mitochondrial membrane and has the capacity to interact with the ER, thus potentially regulating mitochondria-ER interorganellar interactions and exchanges [1, 2]. Several studies have alluded to the importance of ATAD3A in embryonic development. Deletion of *Atad3a* in mice causes embryonic lethality at day E7.5, with growth retardation and defective development of the trophoblast lineage [3]. Knockdown of the *Drosophila* ortholog, *belphegor* (*bor*, *dAtad3a*), results in growth arrest during larval development [1], and the *C. elegans* ortholog is essential for mitochondrial activity and development [4]. RNAi studies of human *ATAD3A* in lung cancer cells have documented increased mitochondrial fragmentation and a decreased co-localization of mitochondria and ER [5].

In humans, the *ATAD3* gene family contains three paralogs that appear to have recently evolved by duplication of a single ancestral gene: *ATAD3A*, *ATAD3B*, and *ATAD3C*. These are located in tandem and map to chromosome 1p36.33 [3, 6]. The major *ATAD3A* isoform, p66, is ubiquitously expressed, whereas the major *ATAD3B* isoform, p67, is expressed during development [6], to a lesser extent postnatally [7, 8] and is upregulated in cancer [9, 10]. *ATAD3C* lacks four exons, suggesting that it may be a pseudogene. The genetic architecture dictated by three highly homologous paralogs predisposes the region to genomic instability and rearrangements generated by non-allelic homologous recombination (NAHR) [11, 12].

To date, the allelic spectrum of *ATAD3A-*associated disease [MIM: 617183] includes null, hypomorph, and antimorph alleles [7, 12–17]. Biallelic deletions mediated by NAHR, most often spanning ~ 38 kb between *ATAD3B* and *ATAD3A* and less frequently ~ 67 kb between *ATAD3C* and *ATAD3A*, lead to an infantile-lethal presentation including respiratory insufficiency, neonatal seizures, congenital contractures, corneal clouding and/or edema, pontocerebellar hypoplasia, and simplified sulcation and gyration [7]. Deletions between *ATAD3B* and *ATAD3A* lead to a fusion transcript under the regulation of the weaker *ATAD3B* promoter and thus show decreased expression of an ATAD3B/ATAD3A fusion protein that presumably is sufficient for fetal development but apparently cannot sustain life beyond the neonatal period [7]. The reciprocal, NAHR-mediated duplication at this locus (between *ATAD3C* exon 7 and the homologous *ATAD3A* exon 11, reciprocal to the *ATAD3C-ATAD3A* deletion described by Harel et al. [12]) results in a fusion gene encoding a protein with multiple alterations at key functional residues [18, 19]. We previously hypothesized that the fusion protein acts through a dominant negative mechanism [18]. Homozygosity for presumed hypomorphic missense alleles (p.Thr53Ile, p.Thr84Met) leads to bilateral cataracts, hypotonia, ataxia, and cerebellar atrophy [12, 20]. Finally, a recurrent de novo heterozygous missense variant (p.Arg528Trp) acts as an antimorph or dominant-negative allele and gives rise to a phenotypic spectrum including developmental delay, hypotonia, optic atrophy, axonal neuropathy, and hypertrophic cardiomyopathy [12].

We report on the clinical and molecular findings of 13 individuals from 8 families with biallelic variants at the *ATAD3A* locus and expand the allelic spectrum to include those with a missense variant inherited in *trans* to an expected loss-of-function (deletion or frameshift) allele. In vivo, functional studies for the missense and non-frameshift indel variants in *ATAD3A* using a *Drosophila* model revealed that these were hypomorphic alleles that exhibited diverse allelic strength and shed light on genotype-phenotype correlations.

## Methods

### Exome analysis

Following informed consent, exome sequencing (ES) was pursued on DNA extracted from the whole blood of affected individuals from each of 8 unrelated families (Additional file 1: Table S1). The study design was adapted to each family and was either proband-only (families 2 and 4), trio (parents and affected child in families 1, 3, 6, and 7), or sibship analysis (two and three affected siblings in families 5 and 8, respectively). Family 4 was previously published [21]. For families 2, 5, and 6, samples were ligated before hybridization with the Agilent SureSelect All Exon capture kit (v4, v5, or v6) or Agilent SureSelect Focused exome kit (Santa Clara, CA, USA). Paired-end 100-bp reads were sequenced on a HiSeq 2500 (Illumina, San Diego, CA, USA) or paired-end 150-bp reads on the NextSeq500 (Illumina, San Diego, CA, USA). Sequencing yielded at least 60 million reads with >80× mean coverage and > 98% of target bases at ≥20×. The Illumina HiSeq FASTQ sequencing reads were aligned to reference GRCh37/hg19 by using BWA-MEM (v0.7.12), converted to BAM format file, and subjected to duplicate removal by using Picard (v1.129). GATK (v3.4-46) was used for indel realignment, variant calling, and quality filtering. Variants were filtered as in Stals et al. [21]. Family 3 was sequenced at GeneDx. Briefly, exonic regions and flanking splice junctions of the genome were captured from the genomic DNA and sequenced on an Illumina system with 100 bp or greater paired-end reads. Reads were aligned to reference GRCh37/hg19 and analyzed for sequence variants via custom-developed analysis tools. CNV calling was done by analyzing exome read-depth data. Additional details of capture and data analysis were similar to those previously described for other samples [22]. For families 1, 7, and 8, ES of the affected individual was performed using the Agilent SureSelect All Exon V6 kits (Agilent) for exome enrichment and a NovaSeq6000 (Illumina) platform for sequencing. Reads were aligned to the human reference genome (UCSC hg19), with Burrows-Wheeler Aligner (BWA, V.0.7.8-r455). The average coverage was >100× in all samples, and more than 97% of the targeted regions were covered more than 20×. High-quality indel and single nucleotide variant calling and annotation were performed using GATK v3.1 as well as SAMtools v.0.1.7 using standard filtering criteria. Copy number variations (CNVs) were detected with ExomeDepth and Pindel. Variants were prioritized by searching for homozygous and compound-heterozygous variants with a minor allele frequency < 0.1% in the in-house database comprising > 20,000 exome datasets as well as de novo variants with a minor allele frequency < 0.001%.

### Sanger validation and segregation of the variants

Single nucleotide variants (SNV) of interest were confirmed by Sanger sequencing, and segregation of variants was carried out in available family members. For family 8, segregation was based on SNP arrays (Illumina Global Screening Array).

### Evaluation of CNV boundaries and breakpoint junction analysis

Breakpoint junctions were amplified with either TaKaRa LA Taq (Clontech) or DreamTaq Green PCR Master Mix (Thermo Scientific) with the primers listed in Additional file 2: Table S2. Resultant fragments were separated by agarose gel electrophoresis and sequenced by Sanger dideoxy nucleotide sequencing. Due to the NAHR, most breakpoints at this locus cannot be determined at the base pair resolution; rather, coordinates with 100% identity between the *ATAD3* paralogs are provided as boundaries (Additional file 2: Table S2).

### 3D modeling of protein structure

The 3D model was predicted with I-TASSER [23]. After inspection of the predicted model, two helices (residues 225-242 and 247-264) were inserted manually. The spatial arrangement of the secondary structures was adjusted manually to ensure proper domain separation by the mitochondria inner membrane.

### Cloning and transgenesis

*dAtad3a-T2A-Gal4* allele was generated using modified methods of CRISPR/Cas-9-mediated genome editing [24] and homology-dependent repair [25] by WellGenetics Inc., Taiwan. We targeted the first coding intron of *dAtad3a* using gRNA (TGTGATAGCGTGGCGCATGC [CGG]). The gRNA was cloned into a U6 promoter plasmid. Cassette T-GEM (1) is composed of an attP site, a linker for phase 1 in-frame expression, T2A, Gal4, Hsp70 transcription terminator, a floxed 3xP3-RFP, and an inverted attP (*attB-splicing acceptor-T2A-Gal4-polyA-loxP-3xP3-RFP-loxP-attB*) [26]. The cassette and two homology arms were cloned into pUC57-Kan as a donor template for repair. The cassette contains an upstream homology arm (HA_L_ - 1016 bp) and a downstream homology arm (HA_L_ - 1039 bp). The homology arms were amplified using the following primers: HA_L_-F: 5′-GCACGCCCACAATTAGCATT-3′, HA_L_-R 5′-GGTTATGCAATTGGCTGATGAAA-3′; HA_R_-F: 5′-GGAGGCCCTCGAGCTGTC-3′, HA_R-_R: 5′-CCAGTCGAACACCTTGTGGA-3′. The gRNA and hs-Cas9 were supplied in DNA plasmids, together with donor plasmid for microinjection into the embryos of control strain *w*^*1118*^. F1 progenies carrying a selection marker of 3xP3-RFP were further validated by genomic PCR and Sanger sequencing. 3xP3-RFP was removed by Cre recombinase.

For construction of pUASTattB-dAtad3a^WT^-V5, a full-length dAtad3a cDNA was amplified by PCR from a pUAST-dAtad3a clone [12] and then subcloned into a pUASTattB vector [27] using the following primers: 5′-GGATCCaaa**ATG**TCGTGGCTTTTGGGCAGG-3′, 5′-GCGGCCGC**TTA**GGTGCTATCCAGTCCGAGCAGTGGATTCGGGATCGGCTTGCCGCCGCTTCC CAGTTTCTTTGCAGTTAGGGTG-3′.

pUASTattB-dAtad3a^L83V^-V5, pUASTattB-dAtad3a^F56L^-V5, pUASTattB-dAtad3a^R176W^-V5, pUASTattB-dAtad3a^G242V^-V5, pUASTattB-dAtad3a^R333P^-V5, and pUAStattB-dAtad3a^K574del^ were generated by site-directed mutagenesis PCR using the following primers: (L83V)-F: 5′-cacgcccgggaggccctcgagGTGtccaagatgcaggaggccacc-3′, (L83V)-R: 5′-ggtggcctcctgcatcttggaCACctcgagggcctcccgggcgtg-3′; (F56L)-F: 5′-aaggccatggaagcgtaccgcTTAgatTCGTCGGCGCTGGAACGT-3′, (F56L)-R: 5′-ACGTTCCAGCGCCGACGAatcTAAgcggtacgcttccatggcctt-3′; (R176W)-F: 5′-gtccagcgtcaagaggccatgTGGcgccagaccatcgagcacgag-3′, (R176W)-R: 5′-ctcgtgctcgatggtctggcgCCAcatggcctcttgacgctggac-3′; (G242V)-F: 5′-gctggtactgttatcggtgccGtTgctgaggctatgcttaccgac-3′, (G242V)-R: 5′-gtcggtaagcatagcctcagcAaCggcaccgataacagtaccagc-3′; (R333P)-F: 5′-ctaaatccgaagctggaggaaCcGcttcgtgacattgccatcgcc-3′, (R333P)-R: 5′-ggcgatggcaatgtcacgaagCgGttcctccagcttcggatttag-3′; and (K574del)-F: 5′-gctgctcagcagcataagcagatggcctggctttcggatcaggag-3′, (K574del)-R: 5′-ctcctgatccgaaagccaggccatctgcttatgctgctgagcagc-3′. A series of pUASTattB-dAtad3a constructs were injected into *y,w,ΦC31; VK37* embryos, and transgenic flies were selected.

### Fly strains and maintenance

The following stocks were obtained from the Bloomington Drosophila Stock Center at Indiana University (BDSC): *w*^*1118*^*; PBac {PB}bor*^*c05496*^*/TM6B, Tb*^*1*^, *w*^*1118*^*; Df (3R)Excel7329/TM6B, Tb*^*1*^*,* and w*; 20xUAS-IVS-mCD8::GFP (on III). All flies were maintained at room temperature (21 °C). All crosses were kept at 25 °C.

### Western blotting

Fly heads were homogenized in 1× Laemmli sample buffer containing 2.5% β-mercaptoethanol (Sigma-Aldrich). After boiling for 10 min, the samples were loaded into 4–20% Mini-PROTEAN® TGX Stain-Free™ Protein Gels (Bio-Rad), separated by SDS-PAGE, and transferred to the nitrocellulose membranes (Bio-Rad). The primary antibodies were used for overnight shaking at 4 °C by the following dilution: mouse anti-V5 (Invitrogen Cat# R960-25 RRID: AB_2556564), 1:2000; mouse anti-ATP5A (Abcam Cat# ab14748 RRID: AB_301447), 1:2000; mouse anti-Actin (MP Biomedicals Cat# 8691002), 1:20,000; and rabbit anti-Ref2(P) (kindly provided by Sheng Zhang). HRP-conjugated goat anti-rabbit (Invitrogen Cat# G-21234 RRID: AB_2536530) and anti-mouse (Invitrogen Cat# A-28177 RRID: AB_2536163) were used at 1:7000 and visualized with ECL (Bio-Rad).

### Embryo collection and immunostaining

Embryos were collected on grape juice plates for 24 h at  25°C. Collected embryos were washed twice with deionized water and dechorionated with 50% bleach for 3 min. After rinsed thoroughly, embryos were fixed for 30 min by 1:1 ratio of Heptane (Sigma Aldrich Cat #246654-1L) and 4% formaldehyde (Thermo Fisher Cat#F79500) in 1× phosphate-buffered saline (PBS), pH 7.4. To remove the vitelline membranes, embryos were shaken vigorously in methanol 5 times. 1× PBS, pH 7.4 containing 0.2% BSA and 0.3% Triton-X100 were used for rehydration and washing. The primary antibodies were used for overnight at the following dilutions: rat anti-Elav 1:500 (DSHB Cat# 7E8A10 RRID:AB_528218), mouse anti-ATP5A 1:500 (Abcam Cat# ab14748 RRID: AB_301447), rabbit anti-β-galactosidase 1:250 (Invitrogen Cat# A-11132 RRID: AB_221539), rabbit anti-GFP 1:1000 (Invitrogen Cat# A-11122 RRID:AB_221569), and Alexa 647-conjugated goat anti-Horseradish Peroxidase 1:500 (Jackson ImmunoResearch Labs Cat# 123-605-021 RRID: AB_2338967). Alexa 488-conjugated anti-rat (Invitrogen Cat# A-21208 RRID: AB_2535794), Alexa 488-conjugated anti-mouse (Invitrogen Cat# A-21202 RRID: AB_141607), and Alexa 568-conjugated anti-rabbit (Invitrogen Cat# A-11011 RRID: AB_143157) secondary antibodies were used at 1:500. Samples were mounted in Vectashield (Vector Labs Cat# H-1000, Burlingame, CA). Imaging was performed using the LSM710 confocal microscope (Zeiss). Images were processed with the Zeiss LSM Image Browser and Adobe Photoshop.

### Quantification of mitochondrial size and numbers

Embryos were collected, stained, and imaged as described above. z-stacks of × 63 confocal images were acquired at a resolution of 1024 × 1024 pixels with 1-μm inter-stack interval and then processed using the Imaris software (Bitplane AG). The volume of the mitochondria was measured using the “Surface” function. Three 200 pixel × 200 pixel × 6 z-stacks cuboid area of VNC (total 7177.73 μm^3^) were selected in each image to measure the mitochondrial volume and number. Signals of oversaturation were excluded manually for precise volume measurements. The numbers of mitochondria were counted and divided by the observed area (μm^3^).

### Adult *Drosophila* thorax sectioning

Flies were fixed in 4% formaldehyde with PBS containing 0.3% TritonX-100 for 4 h at 4 °C on a rotator and rinsed with PBS to remove any residual formaldehyde. Fixed flies were then dissected in PBS. Briefly, fly wings were removed carefully without tearing the tissue of the thorax. Then, the head and abdomen together with the intestines were removed so only the thorax would remain. Holding onto the legs as support to stabilize the thorax, a sharp blade was used to make a slice down the middle of the thorax (dorsal side). The thorax was transferred into an Eppendorf tube and washed with PBS to remove any debris. The primary antibodies were used at the following dilutions: mouse anti-ATP5A 1:500 (Abcam Cat# ab14748 RRID: AB_301447) and rabbit anti-Ref2p 1:1000. Alexa 488-conjugated and Alexa 568-conjugated secondary antibodies were used at 1:500. Samples were mounted in Vectashield. Imaging was performed using the LSM710 confocal microscope (Zeiss). Images were processed with the Zeiss LSM Image Browser and Adobe Photoshop.

### *Drosophila* flight assay

The method was adapted from Pesah et al. [28]. Flies were anesthetized and allocated into individual food vials for 24 h at 25 °C before the assays were performed to allow full recovery from the effects of CO_2_. Each individual vial was inverted into a 500-mL measuring cylinder and gently taped to dislodge the fly into the cylinder. Flies either fell to the bottom in a straight line or flew to the side of the cylinder. Recording of the whole process was undertaken, and analysis was done based on each behavior the flies exhibited. Twenty-five flies of each genotype were assayed.

### *Drosophila* climbing assay

The method was adapted from Madabattula et al. [29]. Twenty-five flies were anesthetized using CO_2_ and allowed to rest in fresh food vials 24 h at 25 °C prior to the assay. Male and female flies were kept separately as gender differences on behavior might be significant. To prepare the climbing apparatus, a distance of 8 cm was measured from the bottom surface of an empty polystyrene vial and marked by drawing a line around the entire circumference of the vial. Flies were transferred without using CO_2_ into different climbing apparatus for each genotype to prevent cross-contamination. The apparatus was closed off by vertically joining it to another empty polystyrene vial using tape, and the flies were left to acclimatize to the surrounding for at least 10 min. Then, the apparatus was gently tapped five times to displace the flies to the bottom of the apparatus, and a video was recorded for 20 s to measure the number of flies able to cross the height of 8 cm at each time point. After a 10-min rest, the assay was repeated. Three trials were conducted.

### Transmission electron microscopy

*Drosophila* thoraxes were imaged following standard electron microscopy procedures using a Ted Pella Bio Wave processing microwave with vacuum attachments. Briefly, the whole thorax with wings was dissected at room temperature in modified Karnovski’s fixative in 0.1 M sodium cacodylate buffer at pH 7.2 and subsequently fixed overnight to 3 days in the same fixative. The pre-fixed thoraxes were then irradiated and fixed again, followed by 3× Millipore water rinses, post-fixed with 1% aqueous osmium tetroxide, and 1% potassium ferrocyanide mixture in Millipore water. This was followed by 3× Millipore water rinses. Ethanol concentrations from 30 to 100% were used for the initial dehydration series, followed by 100% propylene oxide as the final dehydrant. Samples were gradually infiltrated with 3 ratios of propylene oxide and Embed 812, finally going into 3 changes of pure resin under vacuum. Samples were allowed to infiltrate in pure resin overnight on a rotator. The samples were embedded into flat silicone molds and cured in the oven at 62 °C for at least 3 days. The polymerized samples were thin-sectioned at 48–50 nm and stained with 1% uranyl acetate for thirteen minutes followed by 2.5% lead citrate for two and a half minutes the day before TEM examination. Grids were viewed in a JEOL 1400 Plus transmission electron microscope at 80 kV. Images were captured using an AMT XR-16 mid-mount 16 M-pixel digital camera in Sigma mode. Images were contrast adjusted in ImageJ.

## Results

### Clinical reports

**Fig. 1:**
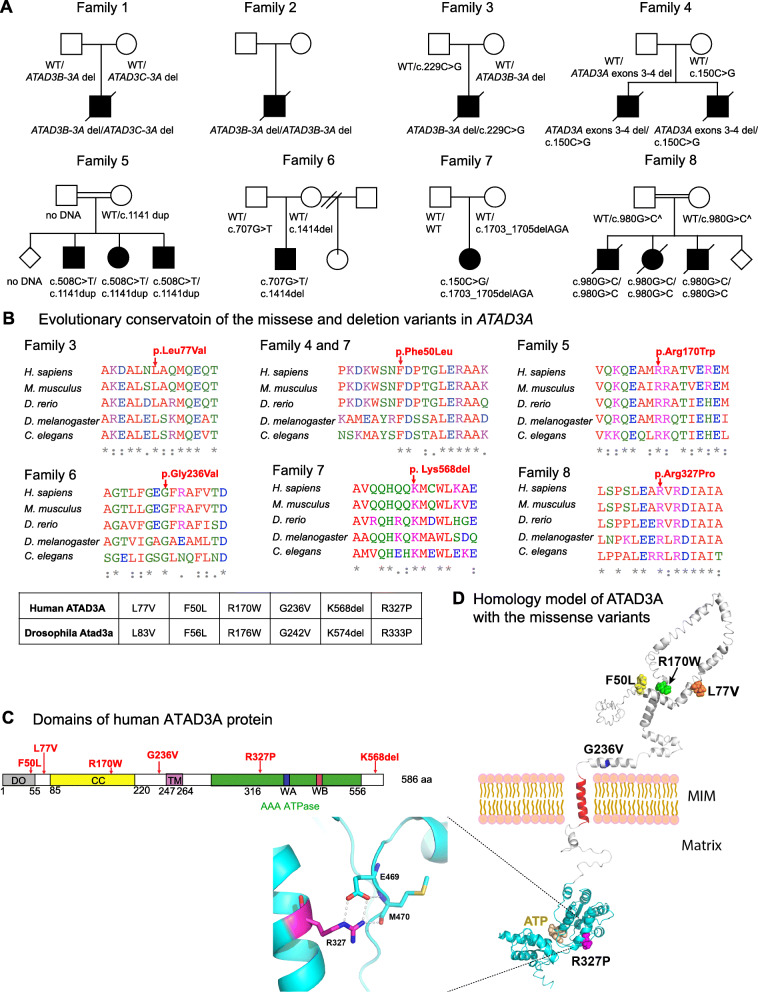
Identification of patients with neurological phenotypes with variants in *ATAD3A*. **a** Pedigrees of studied families, indicating biallelic variants in *ATAD3A* identified in 13 individuals from 8 families. Biallelic deletions were identified in families 1 and 2, loss-of-function alleles (intergenic CNV, intragenic CNV, or frameshift SNV) inherited in *trans* to a missense variant in families 3–7, and a homozygous missense variant in family 8. ^Parents were tested by indirect segregation analysis using SNP arrays. **b** Protein sequence alignment in multiple species confirms evolutionary conservation of p.L77V, p.F50L, pR170W, p.G236V, p.K568del, and p.R327P, in both humans and *Drosophila*. A box shows human SNVs in *ATAD3A* and the *Drosophila dAtad3a* variants homologous to the human variants. **c** Schematic representation of protein domains of human ATAD3A and positions of the SNVs. CC indicates coiled-coil domain. TM indicates putative transmembrane domain. Green indicates AAA+ domain containing Walker A motif (WA) and Walker B motif (WB). **d** In silico protein structure prediction of ATAD3A shows the position of mutated residues

Biallelic variants in *ATAD3A* were identified in 13 individuals from 8 families (Fig. 1a). Family 4 was previously published in Stals et al. [21] and is described in greater molecular detail herein, including breakpoint junction analysis of the two-exon deletion. The biallelic variants identified included different combinations of copy number variants (CNV) and/or single nucleotide variants (SNV). Detailed clinical presentations are supplied in Table 1 and Additional file 1: Table S1. Briefly, individuals with biallelic deletion CNVs showed a neonatal-lethal presentation with respiratory failure, generalized hypotonia, seizures, congenital contractures, bilateral ophthalmologic findings, and brain anomalies, consistent with the reported phenotype [7]. Individuals with a loss-of-function allele (intergenic CNV, intragenic CNV, or frameshift SNV) inherited in *trans* to a missense variant (families 3–6) presented with varied severity of the phenotype (Table 1 and Additional file 1: Table S1), which we hypothesized to correlate with the degree of pathogenicity of the SNV alleles. Finally, two families with other combinations of biallelic variants (non-frameshift indels or missense variants, families 7–8) presented with *ATAD3A-*associated features such as cataracts and hypertrophic cardiomyopathy. Affected individuals in family 8 exhibited severe phenotypes and lethality at 6–7 months after birth. For family 8, indirect segregation based on SNP arrays showed homozygosity of a shared haplotype in the affected siblings and heterozygosity for this haplotype in both parents (Additional file 2: Figure S1). Of note, in family 7 (of German non-consanguineous origin), the c.150C>G; p.(Phe50Leu) was de novo in the proband and presumably arose on the paternal allele, since the c.1703_1705delAGA variant was inherited from the mother (Additional file 2: Figure S2). Identity by state (IBS) analysis from exome sequencing data showed that the index patient and father share 52% of alternative alleles, excluding non-paternity. Due to the complexity of the genomic region surrounding the c.1703_1705delAGA and no available RNA from the proband, we were unable to phase the variants in family 7. However, an identical SNV (c.150C>G; p.(Phe50Leu)) was identified in family 4 (non-consanguineous Romanian descent) where it was inherited from an unaffected mother in *trans* to a paternal 2-exon deletion in *ATAD3A.* This supported the notion that the c.150C>G; p.(Phe50Leu) variant leads to recessive inheritance rather than dominant inheritance and that it probably functions as a hypomorph variant rather than as a gain-of-function or a dominant negative variant. *Drosophila* studies were undertaken to provide further functional support for variants of interest.

**Table 1:** Summary of significant clinical findings

	Family 1	Family 2	Family 3	Family 4 (2 siblings)	Family 5 (3 siblings)	Family 6	Family 7	Family 8 (3 siblings)
*ATAD3A* variants (NM_001170535.1)	*ATAD3C-ATAD3A* del (~ 67 KB); *ATAD3B-ATAD3A* del (~ 38 kb)	*ATAD3B-ATAD3A* comp het del (~ 38 kb; ~ 38 kb)	*ATAD3B-ATAD3A* del (~ 38 kb); c.229C>G; p.(Leu77Val)	Exon 3–4 del (c.(282+1_283-1)_(444+1_445-1)del c.150C>G; p.(Phe50Leu)	c.1141dup p.(Val381Glyfs*17) c.508C>T, p.(Arg170Trp)	c.1414del p.(His472fs) c.707G>T, p.(Gly236Val)	c.150C>G, p.(Phe50Leu); c.1703_1705delAGA, p.(Lys568del)	c.980G>C, p.(Arg327Pro) (hom)
Age at last exam	13 d (deceased)	30 h (deceased)	19 mo (deceased at 2 y)	Died shortly after birth	17–19 years	3 mo	15 y	6–7 mo (all deceased)
Developmental delay	NR	NR	Global DD	NR	Moderate-severe learning difficulties	NA	Mild DD	NR
Neurological exam	Hypotonia, no respiratory effort	Hypotonia, no respiratory effort	NA	Hypotonia, no respiratory effort	Ataxia, muscle wasting	Central hypotonia, increased peripheral tone	Mild hypotonia	NA
Congenital cataract	+	Cloudy corneas	+	Cloudy corneas (one sibling)	One of 3 siblings	–	+	+
Hypertrophic cardiomyopathy	+	+	+	+	–	+	+	+
Cerebellar atrophy/hypoplasia	+	+	NA	+	+	+	+	NA
Elevated 3-methylglutaconate in urine	NA	NA	NA	+	NA	NA	NA	+
Others	–	Undescended testes	–	–	Hearing loss	–	GH deficiency	–

*Abbreviations*: *DD* developmental delay, *del* deletion, *dup* duplication, *GH* growth hormone, *hom* homozygous, *mo* months, *NA* not available, *NR* not relevant, *y* years

### Breakpoint junction analysis of intergenic and intragenic CNVs

The proband in family 1 had a paternally inherited *ATAD3B-ATAD3A* deletion, encompassed by a larger, maternally inherited *ATAD3C-ATAD3A* deletion (Additional file 2: Figure S3, S4). The former was resolved by breakpoint junction sequencing (Chr1[GRCh37]: g.(1413926_1414584)_(1452593_1453251)del), revealing NAHR as the underlying mechanism. The proband in family 2 had two deletions, both spanning between *ATAD3B* and *ATAD3A* and mediated by NAHR: Chr1:(GRCh37):g.(1416206_1416369)_(1454260_1454423) del and Chr1:(GRCh37):g.(1420558_1420661)_(1458286_1458389) del (Additional file 2: Figure S5, S6, S7). Parental samples were not available, yet breakpoint junction sequencing clearly indicated that these deletions must be in *trans*. The proband in family 3 had a paternally inherited SNV inherited in *trans* to a maternally inherited *ATAD3B-ATAD3A* deletion, again resolved at base-pair resolution: Chr1:(GRCh37):g.(1416206_1416369)_(1454260_1454423) del (Additional file 2: Figure S8, S9). Interestingly, the two affected individuals in family 4 had a maternally inherited SNV in *trans* to a paternally inherited intragenic deletion of exons 3 and 4. This would lead to an in-frame deletion of 162 nucleotides or 54 highly conserved amino acids (Additional file 2: Figure S10) in the coiled-coil domain of the protein. Breakpoint junction analysis revealed the proximal breakpoint at chr1:1451952[hg19], within an AluSx1 element, and the distal breakpoint at chr1:1453386[hg19] within a (CACG) n simple repeat. Sequencing of the breakpoint junction revealed microhomology of four nucleotides (Additional file 2: Figure S10), implicating fork stalling and template switching (FoSTeS) or microhomology-mediated break-induced replication (MMBIR) as the underlying mutational mechanism. This is in contrast to the NAHR-mediated CNVs frequently encountered at this locus. Additional file 2: Figure S11 summarizes the various intergenic and intragenic deletions identified in this study.

### In silico analysis of SNVs identified in affected individuals

The five *ATAD3A* missense and one non-frameshift indel variants identified in affected individuals include (provided according to NM_001170535.3, see Additional file 2: Table S3): c.150C>G, p.(Phe50Leu), c.229C>G, p.(Leu77Val), c.508C>T, p.(Arg170Trp), c.707G>T, p.(Gly236Val), c.1703_1705delAGA (Lys568del), and c.980G>C, p.(Arg327Pro). These variants will be referred to as F50L, L77V, R170W, G236V, K568del, and R327P, respectively. The variants have not been reported previously as pathogenic variants and were not seen in the homozygous state in gnomAD, the largest available population database (https://gnomad.broadinstitute.org/) [30]. All of the altered residues are evolutionarily conserved in species of the animal kingdom (Fig. 1b). Notably, the p.(Leu77Val) variant (family 3) affects the same amino acid as a previously reported variant p.(Leu77Arg) [13]. In both cases, the variant was inherited in *trans* to a deletion or stopgain variant, and resulted in a severe clinical presentation including congenital cataract or corneal clouding, brain abnormalities, and seizures. This further supports the pathogenicity of the p.(Leu77Val) variant.

In silico structural modeling of ATAD3A by I-TASSER [23] and secondary structure prediction by PredictProtein [31] suggested that each of the variants would alter the predicted protein structure (Fig. 1d). Phe50 is located next to the first α-helix following the disordered region. Leu77, Arg170, Gly236, and Arg327 are strictly conserved and found in α-helices. Leu77 is predicted to be buried inside the ATAD3A structure and is not exposed at the surface. The p.(Leu77Val) variant could affect a hydrophobic interaction between the α-helix and another part of the structure due to the shortening of the side chain by one carbon. On the contrary, Arg170 is predicted to be surface exposed. Hence, the p.(Arg170Trp) variant may increase surface hydrophobicity, reducing solubility and resulting in a less stable protein. Gly236 is located next to the GxxFG motif that guides the folding and assembly of the membrane-spanning amphipathic α-helix. The p.(Gly236Val) variant may affect the interaction between this potential transmembrane helix and the mitochondrial inner or outer membrane or between neighboring transmembrane helices of the ATAD3A hexamer due to the longer side chain. Arg327 is located next to the ATP binding pocket. The side chain of Arg327 forms a salt bridge with the carboxylate of the Glu469 side chain and also makes a hydrogen bond interaction with the main chain carbonyl of Met470 (Fig. 1d). This interaction would be abolished by the p.(Arg327Pro) variant, resulting in structural changes that could impact ATP binding. K568 is a part of the long helix near the C-terminus which is solvent-exposed toward the C-terminus of the helix. K568 is followed by 4 hydrophobic residues of which 3 are predicted to be part of the helix. In addition to losing one charged residue, deletion of K568 may also cause structural disruption of the helix and the following loop and expose those hydrophobic residues to the solvent, resulting in low protein stability. Collectively, in silico analyses suggest that these variants could impair ATAD3A function.

### Generation of a null *Drosophila dAtad3a* allele for complementation studies

**Fig. 2:**
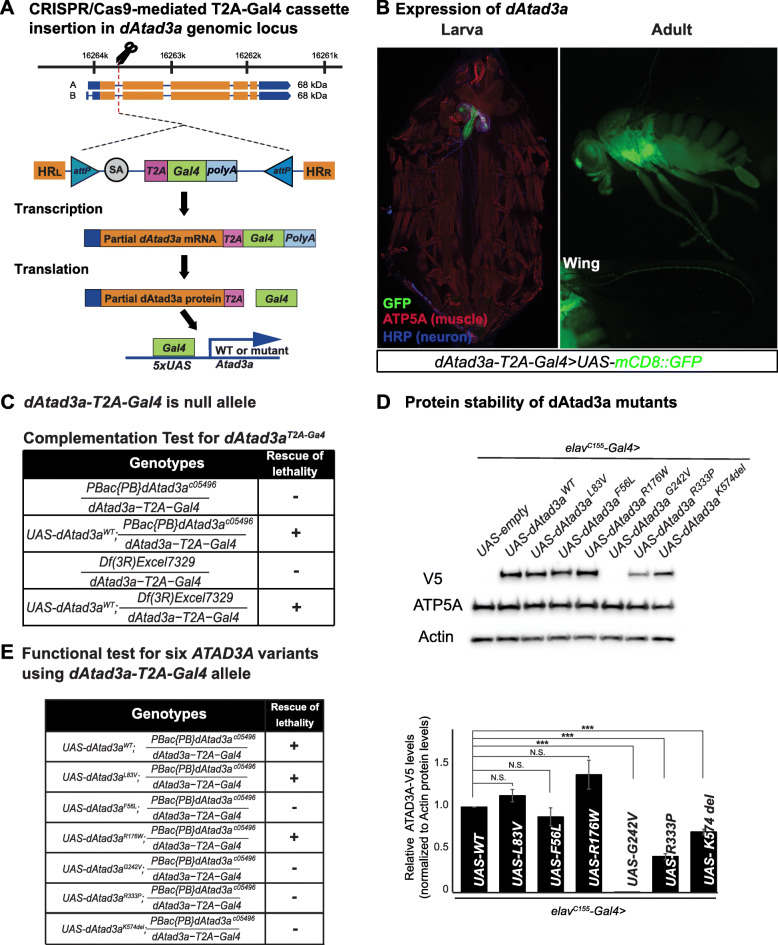
*Drosophila* *Atad3a* models show various strength of *ATAD3A* missense variants. **a** A schematic of the generation of *dAtad3a-T2A-Gal4* by CRISPR-Cas9 gene editing and the translation of a Gal4 protein by a ribosomal skipping mechanism. The location of the *attP-SA-T2A-Gal4-polyA-attP cassette* insertion into the *dAtad3a* genomic locus is indicated by the dotted lines. The T2A-Gal4 cassette consists of a splice acceptor (SA, light gray) followed by a ribosomal skipping T2A peptide sequence (pink), a Gal4 coding sequence (green), and a polyadenylation signal (light blue). Two inverted *attP* sites (blue) are positioned at the 5′- and 3′-end of the cassette. **b** Expression of *UAS-mCD8::GFP* under the control of *dAtad3a-T2A-Gal4* is monitored in larvae and adult flies. **c** Complementation test results of *dAtad3a-T2A-Gal4* alleles. +, complement; −, failure to complement. *dAtad3a-T2A-Gal4* fails to complement a deficiency (*Df (3R)Excel7329*) that lacks the *dAtad3a* locus and *PBac {PB}dAtad3a*^*c05496*^ null allele, which were rescued by the expression of wild-type *dAtad3a* cDNA. These data indicate that *dAtad3a-T2A-Gal4* is a loss-of-function mutant. **d** Western blots for fly heads expressing wild-type dAtad3a-V5 or dAtad3a-V5 carrying the homologous SNV mutations identified from patients. Five replicates were quantified. Error bars indicate SEM. *P* values were calculated using Student’s *t* test. ***P < 0.001. N.S. indicates not statistically significant. **e** The lethality caused by *dAtad3a* loss was rescued by the expression of wild-type *dAtad3a*, and *dAtad3a* carrying L83V or R176W but not by those carrying F56L, G242V, K574del, or R333P

To investigate the functional consequences of SNVs in *ATAD3A*, we created a new *Drosophila Atad3a* (*dAtad3a*) allele based on recently developed CRISPR/Cas-9-mediated genome editing and *Drosophila* genetic technologies [24–26]. To create a null allele, we introduced an artificial exon cassette carrying *attP-SA (splicing acceptor)-T2A-Gal4-polyA-attP* into the first coding intron of *dAtad3a* (referred to as *dAtad3a-T2A-Gal4*) (Fig. 2a). These flies produce an N-terminal portion of the dATAD3A protein as well as the Gal4 protein whose expression is under control of endogenous *cis*-elements of *dAtad3a*. Gal4 is a transcriptional activator that drives the expression of transgenes by binding upstream activating sequence (UAS) (Fig. 2a). To test the expression patterns of *dAtad3a*, we generated flies having a *dAtad3a-T2A-Gal4* allele with *UAS-mCD8::GFP*. We found that dATAD3A is expressed ubiquitously during embryogenesis and that the expression pattern includes neurons in the brain and ventral nerve cord (VNC) (Additional file 2: Figure S12). dATAD3A remains highly expressed in the brain in both larval and adult stages. Moreover, dATAD3A is expressed in the adult thorax and in the peripheral neurons in adult wings (Fig. 2b).

To test whether the *dAtad3a-T2A-Gal4* allele is a loss-of-function mutation, we performed complementation studies. Flies carrying a *dAtad3a-T2A-Gal4* allele and a *dAtad3a* loss-of-function allele (*PBac {PB}dAtad3a*^*c05496*^) or fly mutants lacking the entire *dAtad3a* genomic region (*Df (3R)Excel7329*) exhibited lethality in embryo stages (Fig. 2c). The lethality caused by *dAtad3a* loss was fully rescued by the expression of wild-type *dAtad3a* cDNA (*UAS*-*dAtad3a*^*WT*^) (Fig. 2c). Hence, the results indicate that *dAtad3a-T2A-Gal4* is a severe loss of function allele.

### G242V, R333P, and K574del lead to a reduction in dAtad3a protein levels

To determine whether the series of SNVs in *ATAD3A* identified from affected individuals alter in vivo protein levels of ATAD3A, we generated transgenic flies that allow the expression of *Drosophila dAtad3a* cDNA carrying homologous mutations of the human SNVs including L77V, F50L, R170W, G236V, K568del, and R327P (*UAS-dAtad3a*^*L83V*^, *UAS-dAtad3a*^*F56L*^, *UAS-dAtad3a*^*R176W*^, *UAS-dAtad3a*^*G242V*^, *UAS-dAtad3a*^*K574del*^, *UAS-dAtad3a*^*R333P*^) (Fig. 1b, Table) under the control of UAS. We expressed each transgene together with wild-type *dAtad3a* (*UAS-dAtad3a*^*WT*^) using the pan-neuronal Gal4 driver (*elav*^*C155*^*-Gal4*). All transgenes carry a C-terminal V5 tag. Western blot analysis for adult heads revealed that no protein was detected from *dAtad3a*^*G242V*^expression, and the protein levels of dAtad3a^R333P^ and dAtad3a^K574del^ were lower than those in wild-type control (dAtad3a^WT^) (Fig. 2d). On the contrary, the protein levels from the other three transgenes (*UAS-dAtad3a*^*L83V*^, *UAS-dAtad3a*^*F56L*^, and *UAS-dAtad3a*^*R176W*^) were not significantly different than protein levels in the wild-type control (*dAtad3a*^*WT*^) (Fig. 2d). Hence, these results indicate that the G242V variant is a protein null allele and that R333P and K574del moderately affect protein levels.

### Severely detrimental variants fail to rescue lethality and neurogenesis defects in *Drosophila*

To determine the effects of the series of SNVs in *ATAD3A* identified from affected individuals on in vivo function of ATAD3A, we tested whether the expression of each variant rescues the developmental lethality caused by *dAtad3a* loss. We found that expression of *dAtad3a*^*F56L*^, *dAtad3a*^*G242V*^, *dAtad3a*^*R333P*^, or *dAtad3a*^*K574del*^ completely failed to rescue the lethality caused by loss of *dAtad3a*, indicating that these four variants are severe loss of function alleles (Fig. 2e). Failure of lethality rescue by G242V is consistent with the Western results showing complete loss of the protein with this mutation (Fig. 2d). The failure of lethality rescue by F56L, R333P, and K574del could result from functional defects rather than the moderately decreased protein levels (~ 60% for R333P, ~ 30% for K574del) because one copy loss of *dAtad3a* (*dAtad3a*^*T2A-Gal4*^*/+* or *PBac {PB}dAtad3a*^*c05496*^*/+*) does not affect viability. On the contrary, expression of *dAtad3a*^*L83V*^ or *dAtad3a*^*R176W*^ fully rescued the developmental lethality caused by *dAtad3a* loss as we obtained adult *dAtad3a* null flies expressing *dAtad3a*^*L83V*^ or *dAtad3a*^*R176W*^ in expected Mendelian ratios (Fig. 2e). In addition, *dAtad3a* mutant larvae expressing *dAtad3a*^*L83V*^ or *dAtad3a*^*R176W*^ exhibited comparable mitochondrial content to those of flies expressing *dAtad3a*^*WT*^ (Additional file 2: Figure S13). These results indicate that the flies carrying L83V and R176W variants did not exhibit developmental defects. Hence, the results indicate that F56L, G242V, R333P, and K574del are severe loss-of-function alleles.

**Fig. 3:**
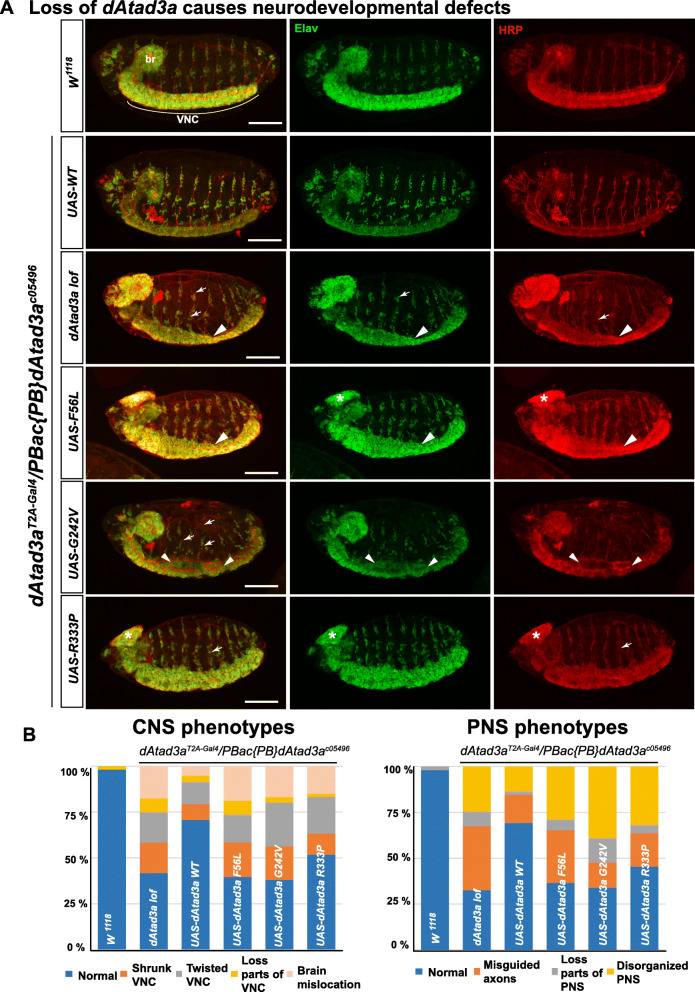
*dAtad3a* lof, F56L, G242V, and R333P variants lead to severe neurodevelopmental defects. **a** Confocal micrographs of wild-type (*w*^*1118*^), *dAtad3a*-null mutant embryos and those expressing *dAtad3a*^*WT*^, *dAtad3a*^*F56L*^, *dAtad3a*^*G242V*^, and *dAtad3a*^*R333P*^. Elav (green)-stained neurons and anti-HRP (red)-stained neuronal membranes. Br indicates brain, and VNC indicates ventral nerve cord. Arrowheads indicate shrunken and twisted VNC. Arrows indicate misguided and loss of neurons in the PNS. Scale bars indicate 100 μm. **b** Quantification of CNS and PNS phenotypes shown in mutant embryos. The numbers of embryos for these analyses are as follows: CNS—*w*^*1118*^ (*n* = 54), wt (*n* = 58), null (n= 96), F56L (*n* = 53), G242V (*n* = 71), and R333P (*n* = 60). PNS—*w*^*1118*^ (*n* = 51), wt (*n* = 52), null (86), F56L (*n* = 52), G242V (*n* = 59), and R333P (*n* = 44)

To investigate the phenotypic strength of F56L, G242V, and R333P, we decided to characterize phenotypes during embryogenesis from *dAtad3a* null mutants as well as each mutant because most animals expressing these variants die before the 1st instar larvae stage. No reports for phenotypes caused by *dAtad3a* loss during embryogenesis have been documented so far. Biallelic deletion of *ATAD3A* and adjacent *ATAD3* paralogs in humans causes severe neuro-developmental defects including congenital pontocerebellar hypoplasia and neonatal death [7, 12] Thus, we sought to determine whether *dAtad3a* loss causes defects in neurodevelopment in *Drosophila* embryos using anti-Elav (a neuronal marker) and anti-HRP (a marker for neuronal membranes) antibodies. We examined stage 15 embryos in which the central nervous system (CNS) including the brains and ventral nerve cord (VNC), and the peripheral neurons and their neuronal projections are well established (Fig. 3a). The *dAtad3a* null mutants exhibit a wide range of neurogenesis defects. We found that loss of *dAtad3a* results in 58% of embryos with defects in CNS development and 67% with defects in peripheral nervous system (PNS) development (*dAtad3a* lof, Fig. 3a). The CNS defects include brain mis-location, twisted and shrunken VNC, and partial absence of the VNC. The PNS phenotypes include the partial absence of PNS cells, misguided PNS neural tracks, and failure of correct specification of the PNS cells. The phenotypes caused by *dAtad3a* loss were significantly rescued by expressing wild-type *dAtad3a* (*UAS-dAtad3a*^*WT*^) (29% CNS defects; 31% PNS defects), but not by expressing *dAtad3a*^*F56L*^, *dAtad3a*^*G242V*^, or *dAtad3a*^*R333P*^ (Fig. 3b). Expression of F56L (60% CNS; 63% PNS) and G242V (62% CNS; 66% PNS) exhibited a phenotype strength comparable to those in null mutants, whereas R333P (48% CNS; 55% PNS) showed slightly weaker phenotypes (Fig. 3b). Finally, the null mutants and three loss-of-function (LOF) alleles exhibited an abnormal increase in mitochondrial content and size, as compared to the null mutant embryos complemented with wild-type *dAtad3a* and as compared to wild-type control embryos (Additional file 2: Figure S14, S15). Hence, we discovered that *dAtad3a* loss leads to severe neurodevelopmental defects in *Drosophila* embryos.

### Variants of mild severity affect locomotion, mitochondrial morphology, and autophagy in *Drosophila*

**Fig. 4:**
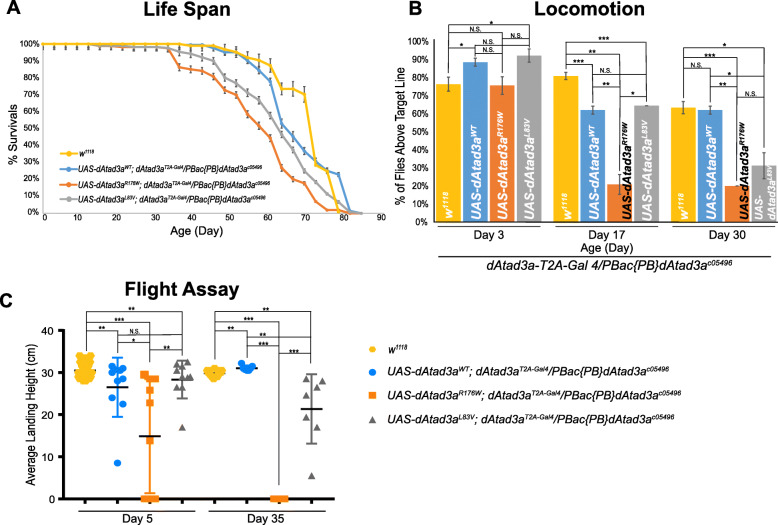
L83V and R175W variants cause behavioral defects in adult flies. **a** *dAtad3a*-null mutant flies expressing L83V, and R176W were short lived compared to wild-type rescue animals and wild-type control (*w*^*1118*^). **b** *dAtad3a*-null mutant flies expressing L83V, and R176W exhibited progressive climbing defects compared to wild-type rescue controls and wild-type control (*w*^*1118*^). **c** *dAtad3a*-null mutant flies expressing R176W exhibited defects in flight ability on the 5th day of their life and complete failure of flight on the 35th day. *dAtad3a* mutant flies expressing L83V exhibited progressive decline of flight ability compared to rescue control and wild-type control (*w*^*1118*^). **b**, **c** Three biological replicates (25 flies per group) were quantified. Error bars indicate SEM. *P* values were calculated using Student’s *t* test. *P < 0.05, **P < 0.01,***P < 0.001. N.S. indicates not statistically significant

To investigate the phenotypes of L83V and R176W, we sought to characterize post-developmental phenotypes such as behavioral and age-associated phenotypes because the expression of these variants did not exhibit developmental defects (Fig. 2e, Additional file 2: Figure S13). First, we performed life span assays and found that *dAtad3a* null flies expressing L83V or R176W exhibited shorter life spans compared to those in *dAtad3a* null flies expressing wild-type *dAtad3a* and wild-type control flies (*w*^*1118*^) (50% survival at day 57 (R176W), day 62 (L83V), day 64 (WT), and day 72 (*w*^*1118*^)) (Fig. 4a). dATAD3A is mainly expressed in the adult thorax and head (Fig. 2b); thus, loss of its function may affect locomotion behavior. To test this, we performed a climbing assay. We found that both variants exhibited age-dependent locomotion defects, and R176W showed more severe locomotion defects compared to L83V flies (Fig. 4b). We also performed a flight assay that is more sensitive than the climbing assay. Flies expressing R176W exhibited a flight defect at a young age (day 5) and failed to fly at all in old age (day35), whereas flies expressing L83V showed normal flight at young age, but mildly defective flight in old ages (Fig. 4c). Collectively, these results indicate that both L83V and R176W variants are partial loss-of-function alleles and that R176W has more defective gene function compared to L83V.

We previously showed that human fibroblasts carrying the de novo variant p.Arg528Trp exhibited an increase in the mitophagic vesicles and expression of *dAtad3a* carrying p.Arg534Trp, the homologous mutation of human p.Arg528Trp, leads to small mitochondria with aberrant cristae as well as an increase in autophagic vesicles in larvae muscles [12]. These findings suggest that aberrant autophagy or mitophagy may underlie the behavioral defects in *dAtad3a* mutant flies expressing L83V or R176W (Fig. 4). To test this, we sought to examine mitochondria morphology and autophagy in adult thorax muscles. First, we assessed mitochondrial morphology using an antibody for ATP5A in both young (5-day-old) and old (21-day-old) adult flies. We found that R176W leads to smaller mitochondria with a rounded shape compared to those in wild-type controls (Additional file 2: Figure S16). On the contrary, the animals expressing L83V exhibited irregular size of mitochondria with slightly longer mitochondria on average (Additional file 2: Figure S16). These results indicate that both R176W and L83V variants affect mitochondrial dynamics, which in turn may lead to increased autophagy or mitophagy.

**Fig. 5:**
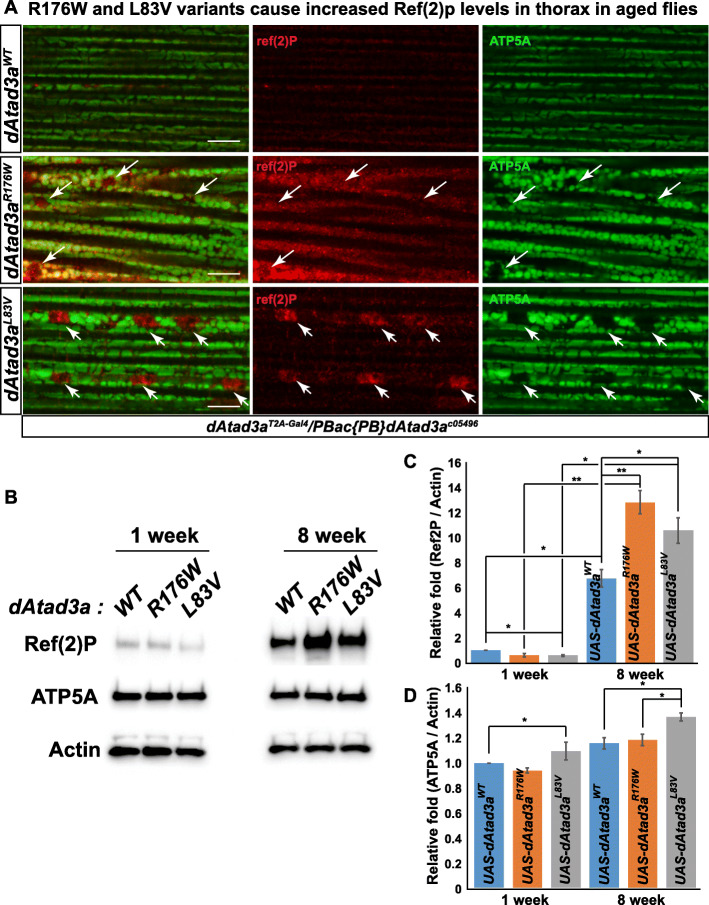
L83V and R176W variants cause increased p62 levels in the thorax in aged flies. **a** Confocal micrographs of the thorax muscle from 8-week-old flies—*dAtad3a*-null mutants expressing *dAtad3a*^*WT*^, *dAtad3a*^*R176W*^, or *dAtad3a*^*L83V*^. ATP5A (green) labels mitochondria. Ref (2) P is the *Drosophila* homolog of p62 (red). Arrows indicate Ref (2) P signals with the absence of ATP5A signals. Scale bars indicate 100 μm. **b** Western blots for the protein levels of Ref (2) P, ATP5A, and Actin from *dAtad3a* mutant fly thoraxes expressing *dAtad3a*^*WT*^, *dAtad3a*^*R176W*^, or *dAtad3a*^*L83V*^ (*n* = 10 per genotype). **c** Quantification of Ref (2) P and **d** ATP5A level. Ref (2) P and ATP5A were normalized by Actin. Three biological replicates were quantified. Error bars indicate SEM. *P* values were calculated using Student’s *t* test. *P < 0.05, **P < 0.01

**Fig. 6:**
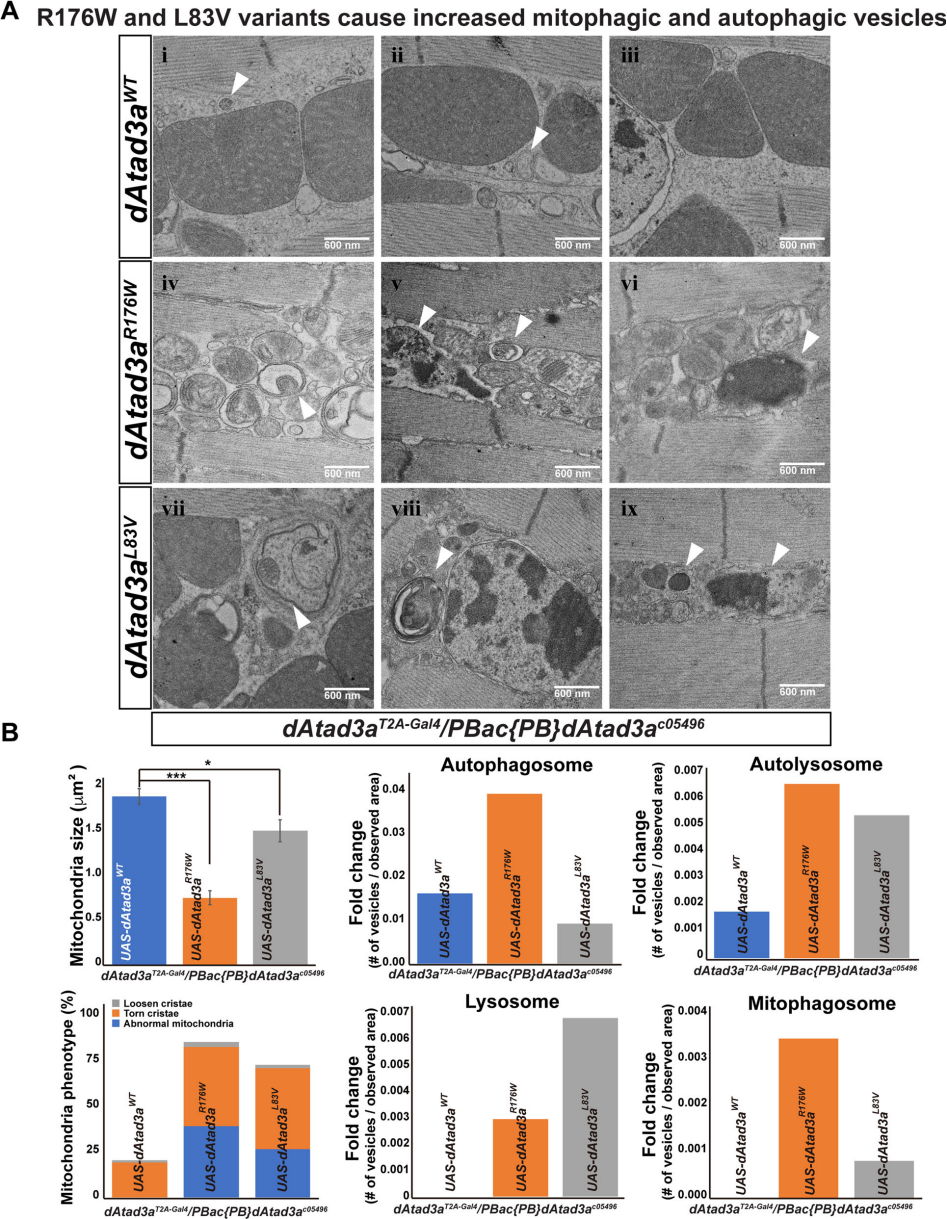
L83V and R176W variants cause aberrant mitochondrial morphology, increased autophagic and mitophagic vesicles. **a** Electron micrographs of the thorax muscles from 8-week-old *dAtad3a* mutant flies expressing *dAtad3a*^*WT*^, *dAtad3a*^*R176W*^, or *dAtad3a*^*L83V*^. Arrows show autophagosomes (i, ii, and v), autolysosomes (v and viii), lysosomes (vi and ix), and mitophagosomes (iv and vii). Scale bars indicate 600 nm. **b** Quantification of mitochondria size, mitochondria phenotypes, numbers of autophagosome, autolysosome, lysosome, and mitophagosome. The respective number of the vesicles was normalized by observed area (μm^2^). Error bars indicate SEM. *P* values were calculated using Student’s *t* test. *P < 0.05, ***P < 0.001

To test whether *dAtad3a* carrying R176W or L83V variants cause an increase in autophagy, we measured the levels of ref(2)P, the *Drosophila* orthologue of p62, an autophagy marker, in adult muscles. While flies expressing R176W or L83V exhibited comparable levels of ref(2)P as compared to wild-type controls as young animals (7-day-old), the older (8-week-old) mutants expressing R176W or L83V, exhibited significantly higher levels of ref(2)P than those in wild-type controls (Fig. 5b). We found that in muscles expressing R176W, most mitochondria marked by ATP5A were co-localized with ref(2)P signals. We also found large vacuole-like structures that were void of ATP5A, but ref(2)P positive in R176W muscles (Fig. 5a, arrows, *dAtad3a*^*R176W*^). In the muscles expressing L83V, we found a patch of higher ref(2)P signals that were completely void of ATP5A (Fig. 5a, arrows, *dAtad3a*^*L83V*^), suggesting that mitochondria with higher ref(2)P underwent mitophagy and were degraded through autophagosomes. To further characterize this, we performed transmission electron microscopy (TEM). TEM in 56-day-old animals revealed that R176W led to small mitochondria and increased autophagic intermediates, whereas wild-type rescue animals exhibited normal mitochondria with lower numbers of autophagic intermediates (Fig. 6a, b). Interestingly, the muscles expressing L83V showed many normal mitochondria (Additional file 2: Figure S17), but parts of the muscles were filled with autophagic intermediates (Fig. 6a, b; Additional file 2: Figure S17), which is consistent with the ref(2)P results (Fig. 5a). In addition, we found that R176W and L83V cause small mitochondria with bar-shaped cristae (Fig. 6a, b) as well as distinctive cristae abnormalities—cristae are loosened and torn apart (Additional file 2: Figure S17). Collectively, the data indicate that both R176W and L83V variants lead to increased autophagy and mitochondria loss and aberrant cristae and that the detrimental effect of dAtad3a^R176W^ is more severe than dAtad3a^L83V^.

### Functional studies in *Drosophila* provide support of pathogenicity

Functional studies for the five missense variants in *Drosophila* were consistent with bioinformatic predictions, where the human variants p.(Leu77Val) and p.(Arg170Trp) had more benign prediction scores as compared to the other missense variants (Additional file 2: Table S3). However, caution must be exercised with bioinformatics prediction scores, as the p.(Phe50Leu) variant also had a relatively low conservation score (GERPrs 1.95) yet was clearly pathogenic when tested in vivo. Indeed, the family with the most mild phenotype, family 5, exhibited the p.(Arg170Trp) allele (the mild variant in the *Drosophila* functional studies) in *trans* to a frameshift variant (c.1141dup; p.(Val381Glyfs*17). On the contrary, homozygosity for the p.(Arg327Pro) allele (a severe variant in the *Drosophila* functional studies) led to a severe, infantile lethal phenotype (family 8). Genotype-phenotype correlations must take into account both alleles—the p.(Phe50Leu) allele was associated with a severe phenotype when inherited in *trans* to an intragenic deletion of two exons (family 4), yet with a mild phenotype when observed with a non-frameshift single amino acid deletion (family 7). Overall, *Drosophila* studies provided support of variant pathogenicity and correlated reasonably well with the severity of the clinical phenotype in humans.

## Discussion

The spectrum of *ATAD3A* variants has thus far focused on monoallelic gain-of-function variants and biallelic loss-of-function variants. We report eight families with biallelic variants, ranging from biallelic CNVs to biallelic SNVs and combinations thereof. The relatively wide phenotypic and genotypic spectrum of *ATAD3A-*associated variation calls for caution in the interpretation of the clinical significance of missense variants. To address this, we systematically analyzed the functional effect of six SNVs identified in affected individuals using *Drosophila* models. We showed that F56L, G242V, R333P, and K574del are severe loss of function alleles which fail to rescue the lethality of *dAtad3a* null mutants, whereas L83V and R176W are mild hypomorph variants which rescued developmental lethality but exhibited behavioral defects in adult flies. The results from *Drosophila* studies correlated with the clinical severity of affected individuals—orthologs of the three more severe loss-of-function alleles (F56L, G242V, and R333P in *Drosophila*, or F50L, G236V, R327P in humans) led to severe phenotypes including hypotonia, global developmental delay, cataracts, cardiomyopathy, and structural brain abnormalities (families 4, 6, and 8). Cardiomyopathy seems to be an expansion of the phenotype associated with biallelic deletions/LOF variants [14]. As noted in the “Results” section, the F50L variant was identified in *trans* to a two exon deletion in family 4, yielding a severe phenotype yet was also identified along with a single amino acid deletion (non-frameshift) in family 7, resulting in a more mild phenotype albeit with cataracts, cardiomyopathy, and cerebellar abnormalities. Although the clinical phenotype in family 7 is overtly consistent with *ATAD3A-*associated disorders, a major limitation of this study was that we could not phase the variants (one inherited, the other de novo) in the proband due to technical issues. In contrast to the “severe” alleles, one of the hypomorphic alleles (R176W in *Drosophila* or R170W in humans), when inherited in *trans* to a frameshift variant, led to a mild phenotype reminiscent of the first family reported with biallelic hypomorphic SNV [12]. This relatively weak variant (Additional file 2: Table S3) may have been overlooked or discarded in variant filtering of the exome; nonetheless, functional modeling combined with a clinical phenotype compatible with the mild range of the *ATAD3A* disease spectrum implicates the variant as probably disease-causing.

ATAD3A belongs to the AAA+ protein family. Members of this family form oligomers and have positive cooperativity in ATP binding and hydrolysis, whereby alteration of subunits (i.e., ATPase deficient mutants) affect the function of the entire protein complex [32]. Previous data from studies in *Drosophila* and in patient-derived fibroblasts with heterozygous variants (i.e., p.Arg528Trp; R528W and p.Gly355Asp; G355D) suggested that both alleles act in a dominant-negative fashion [12, 15], consistent with the location of the mutated residues in the key motifs required for the ATPase activity (Gly355, the Walker A motif [15]; R528, the Sensor 2 motif, personal communication with Sukyeong Lee). Here, we show that six SNVs (five missense variants and one non-frameshift indel) act as hypomorphic or loss-of-function alleles rather than dominant-negative. Of the missense variants, four variants including L77V, F50L, R170W, and G236V are located outside of the AAA+ domain (Fig. 1c). Only Arg327 is located in the AAA+ domain (Fig. 1d), which may impact the conformation of the AAA domain. However, human carriers for Arg327Pro (parents of family 8) are unaffected, suggesting that this allele does not function in a dominant negative manner in humans. Western blot in flies showed that G236V (G242V in *Drosophila*) is a protein null (Fig. 2d), consistent with severe loss of function of G242V in vivo. On the contrary, the other five alleles moderately or do not affect protein levels (Fig. 2d), but rather lead to severe or partial loss-of-function of ATAD3A, suggesting the functional importance of the N-terminal and middle CC domains in ATAD3A. The first 50 amino acids were reported to be important to form contact sites between the mitochondria and the ER membrane [1], implicating that the F50L variant may cause defects in mitochondria-ER communication. The CC domain was shown to bind to Drp1 and oligomerization of ATAD3A leads to Drp1 to the mitochondria via the CC domain, resulting in increased fission [33]. This suggests that R170W (R176W, *Drosophila*), located in the CC domain, leads to small mitochondria with increased autophagosome (Fig. 6b) via an increased interaction with Drp1. Further molecular studies of the pathogenic variants and identification of ATAD3A-interacting proteins will provide insight as to how genetic variants cause the etiology at the molecular and cellular levels.

Previous studies in *Drosophila* and in patient-derived fibroblasts with heterozygous variants (i.e., p.Arg528Trpand p.Gly355Asp) revealed a defect in mitochondrial dynamics, possibly triggering mitophagy and resulting in a significant reduction of mitochondria [12]. Here, we also demonstrated that aged muscles expressing R176W and L83V variants exhibited defective mitochondrial membrane dynamics and increased mitophagic vesicles (Fig. 6). Thus, these findings suggest that proper ATAD3A function is required for homeostasis of mitochondrial dynamics and mitophagy. Interestingly, in *Drosophila* embryonic stages, we found that the null and severe LOF alleles (F56L, G242V, and R333P) exhibited increased mitochondrial content (Additional file 2: Figure S14-S15), which may result from the compensatory mechanisms for abnormal mitochondria.

One mechanism for mitophagy was documented in mouse hematopoietic stem cells, in which increased mitophagy in *ATAD3A-*deficient cells has been attributed to perturbation of Pink1-mediated mitophagy [34]. Abnormal regulation of nutrition and metabolism-sensing machineries such as the mechanistic target of rapamycin (mTOR) could be implicated in the etiology caused by loss of *ATAD3A* as mTOR is a major regulator for autophagy and mitophagy. Indeed, Cooper et al. [15] demonstrated upregulated basal autophagy in patient fibroblasts, associated with mTOR inactivation. In mice, *Atad3a* and mTOR have central functions in the biogenesis of mitochondria during development [2, 3, 35]. The target of rapamycin (TOR) signaling positively regulates mitochondrial activity, and the *Drosophila* paralog of *ATAD3A* (*bor*) is downregulated upon rapamycin-dependent inhibition of TOR signaling pathways [36]. Thus, altered mTOR signaling may also contribute to the pathogenesis of *ATAD3A*-related disorders, and targeting mTOR activity could be a potential therapeutic avenue for alleviating symptoms caused by *ATAD3A* mutations.

In addition to altered mitochondrial dynamics, increased mitophagy, and mTOR inactivation in *ATAD3A-*deficient cells [15, 34], alternative pathogenetic mechanisms for *ATAD3A-*associated disorders have been proposed. These include impaired mtDNA and segregation, and aberrant cholesterol channeling and steroidogenesis [37–39]. mtDNA co-sediments with cholesterol, and both mitochondrial integrity and cholesterol metabolism have been linked to neurodegeneration and cerebellar pathology [7]. Fibroblasts from individuals with biallelic *ATAD3* locus deletions displayed enlarged and more numerous mitochondrial DNA (mtDNA) foci, suggesting that *ATAD3A* deficiency causes localized mtDNA aggregation or impairs its proper distribution. Moreover, fibroblasts demonstrated multiple indicators of altered cholesterol metabolism [7]. The associated disease pathology was proposed to result either from compromised rigidity of the inner mitochondrial membrane with impaired mtDNA segregation subsequent to inadequate cholesterol metabolism or from a shortage of cholesterol products in Purkinje cells. Affected individuals whose fibroblasts exhibited impaired cholesterol metabolism often presented with elevated urine levels of 3-methyglutaconic acid (3-MGA) [7, 12, 18]. Interestingly, *SERAC1* deficiency presents with impaired cholesterol metabolism together with elevated 3-MGA levels [40], suggesting that defective cholesterol metabolism and mitochondrial lipid metabolism may be implicated in increased levels of 3-MGA. Whether manipulating cholesterol metabolism and mitochondrial lipid metabolism may ameliorate ATAD3A pathologies remains to be investigated.

## Conclusions

*Drosophila* has been well established as a powerful genetic model organism [41]. We utilized this model organism to assess functional impacts of various SNVs in *ATAD3A* and showed that the allele severity in *Drosophila* correlates with the phenotypic severity in humans. This study further reiterates the clinical findings associated with *ATAD3A* pathogenic variation, including developmental delay, hypotonia, congenital cataracts, hypertrophic cardiomyopathy, and cerebellar atrophy. We contribute to the growing disease-causing allelic spectrum at the *ATAD3A* locus, which includes biallelic NAHR-mediated deletions and a reciprocal monoalleleic duplication; monoallelic dominant-negative variants, biallelic SNVs, and splice-site variants; and now SNVs in trans to intergenic or intragenic deletion alleles.

## Supplementary Information

**Additional file 1 **: **Table S1**. Clinical information: detailed clinical information of individuals with biallelic variants involving *ATAD3A.*

**Additional file 2 **: **Table S2**. Primers used for breakpoint junction analyses: Primers used to define breakpoint junctions in families 1-4. **Table S3**. Missense variants identified in *ATAD3A:* Bioinformatic predictions of missense variants identified in this study. **Figure S1**. Homozygous variant in Family 8: Visualization of exome sequencing reads showing the homozygous variant c.980G>C, p.(Arg327Pro). **Figure S2**. Segregation analysis in Family 7: Data showing that the c.150C>G variant is de novo*,* whereas the c.1703_1705del variant is maternally inherited. **Figure S3**. Compound heterozygous deletion affecting *ATAD3A* in Family 1: Visualization of exome sequencing read alignments indicating two overlapping deletions inherited in trans. **Figure S4**. Breakpoint junction sequencing of paternally inherited *ATAD3B/ATAD3A* deletion in Family 1: Alignment to *ATAD3B* and *ATAD3A* shows that the breakpoint occurred within a region of identity between the paralogs. **Figure S5**. Read depth analysis of exome sequencing data in Family 2: The compound heterozygous deletion can be appreciated. **Figure S6**. Breakpoint junction sequencing of first *ATAD3B/ATAD3A* deletion in Family 2: Alignment to *ATAD3B* and *ATAD3A* shows that the breakpoint occurred within a region of identity between the paralogs. **Figure S7**. Breakpoint junction sequencing of second inherited *ATAD3B/ATAD3A* deletion in Family 2: Alignment to *ATAD3B* and *ATAD3A* shows that the breakpoint occurred within a region of identity between the paralogs. **Figure S8**. Confirmatory array data from Family 3: The heterozygous deletion can be appreciated in the proband and mother’s samples, but not in the father’s sample. **Figure S9**. Breakpoint junction sequencing of maternally inherited *ATAD3B/ATAD3A* deletion in Family 3: Alignment to *ATAD3B* and *ATAD3A* shows that the breakpoint occurred within a region of identity between the paralogs. **Figure S10**. Breakpoint junction sequencing of paternally inherited 2-exon deletion in *ATAD3A* (Family 4): Delineation of the breakpoint junction by Sanger sequencing spanning the deletion, and evolutionary conservation of the skipped exons. **Figure S11**. Schematic diagram of all CNVs identified in this study: Diagram of the six CNVs studied; five of six were resolved at the breakpoint junction level. **Figure S12**. dAtad3a is expressed ubiquitously in embryos: Confocal micrographs of an embryo expressing GFP protein under the control of *dAtad3a-T2A-Gal4* showed that *dAtad3a* is expressed ubiquitously. **Figure S13**. R176W and L83V did not affect mitochondria content and morphology in larvae muscles: Mitochondria content and morphology of *dAtad3a* mutant muscles expressing *dAtad3a*^*R176W*^, or *dAtad3a*^*L83V*^ are comparable to those in wildtype controls. **Figure S14**. *dAtad3a* null, F56L, G242V, and R333P cause increased mitochondrial content in embryos: Mitochondrial content in *dAtad3a* null mutant embryo and those expressing *dAtad3a*^*F56L*^, *dAtad3a*^*G242V*^, or *dAtad3a*^*R333P*^ is higher than those in wild type control embryos. **Figure S15**. dAtad3a null, F56L, G242V, and R333P cause increased mitochondrial numbers and size in embryos: In embryo VNC, *dAtad3a* null mutant and those expressing *dAtad3a*^*F56L*^, *dAtad3a*^*G242V*^, or *dAtad3a*^*R333P*^ exhibited an increase in the mitochondrial size and numbers compared to those in wild type controls. **Figure S16**. R176W causes small mitochondria in adult muscles: *dAtad3a* mutant muscles expressing *dAtad3a*^*R176W*^ exhibited smaller size of mitochondria compared to those expressing *dAtad3a*^*WT*^, or *dAtad3a*^*L83V*^. **Figure S17**. R176W and L83V cause various defects in mitochondria in adult muscles: *dAtad3a* mutant muscles expressing *dAtad3a*^*R176W*^, or *dAtad3a*^*L83V*^ exhibited small, and membrane-defective mitochondria compared to those expressing *dAtad3a*^*L83V*^.

## Data Availability

Clinical ES and DNA sequencing data for families 2–6 are not publicly available as they were generated in clinical diagnostic labs as part of the patients’ clinical investigations and cannot be shared outside of this service. ES data of families 1, 7, and 8 cannot be shared as this is not compliant with the IRB approval. The variant alleles identified in all families have been deposited on ClinVar, with the following accession numbers: SCV001468306–SCV001468318, https://www.ncbi.nlm.nih.gov/clinvar/variation/432628/ [42], https://www.ncbi.nlm.nih.gov/clinvar/variation/992485/ [43], https://www.ncbi.nlm.nih.gov/clinvar/variation/992486/ [44], https://www.ncbi.nlm.nih.gov/clinvar/variation/992487/ [45], https://www.ncbi.nlm.nih.gov/clinvar/variation/992488/ [46], https://www.ncbi.nlm.nih.gov/clinvar/variation/992489/ [47], https://www.ncbi.nlm.nih.gov/clinvar/variation/992490/ [48], https://www.ncbi.nlm.nih.gov/clinvar/variation/992491/ [49], https://www.ncbi.nlm.nih.gov/clinvar/variation/992492/ [50], https://www.ncbi.nlm.nih.gov/clinvar/variation/992493/ [51], https://www.ncbi.nlm.nih.gov/clinvar/variation/992494/ [52], https://www.ncbi.nlm.nih.gov/clinvar/variation/992495/ [53], and https://www.ncbi.nlm.nih.gov/clinvar/variation/992496/ [54].

## Abbreviations

ATAD3A: AAA-domain containing protein 3A
AAA+ ATPase: ATPases associated with diverse cellular activities
*bor*: *Belphegor*
NAHR: Non-allelic homologous recombination
CNV: Copy number variants
SNV: Single nucleotide variants
dAta d3a: Drosophila Atad3a
UAS: Upstream activating sequence
VNC: Ventral nerve cord
CNS: Central nervous system
PNS: Peripheral nervous system
TEM: Transmission electron microscopy
