# Supplementary material for: Functional interpretation of ATAD3A variants in neuro-mitochondrial phenotypes
Source: Genome Med. 2021 Apr 12;13:55. doi: 10.1186/s13073-021-00873-3 (PMC8042885; doi:10.1186/s13073-021-00873-3)
Supplement: Supplementary file 2 — Additional file 2 : Table S2. Primers used for breakpoint junction analyses: Primers used to define breakpoint junctions in families 1-4. Table S3. Missense variants identified in ATAD3A: Bioinformatic predictions of missense variants identified in this study. Figure S1. Homozygous variant in Family 8: Visualization of exome sequencing reads showing the homozygous variant c.980G>C, p.(Arg327Pro). Figure S2. Segregation analysis in Family 7: Data showing that the c.150C>G variant is de novo, whereas the c.1703_1705del variant is maternally inherited. Figure S3. Compound heterozygous deletion affecting ATAD3A in Family 1: Visualization of exome sequencing read alignments indicating two overlapping deletions inherited in trans. Figure S4. Breakpoint junction sequencing of paternally inherited ATAD3B/ATAD3A deletion in Family 1: Alignment to ATAD3B and ATAD3A shows that the breakpoint occurred within a region of identity between the paralogs. Figure S5. Read depth analysis of exome sequencing data in Family 2: The compound heterozygous deletion can be appreciated. Figure S6. Breakpoint junction sequencing of first ATAD3B/ATAD3A deletion in Family 2: Alignment to ATAD3B and ATAD3A shows that the breakpoint occurred within a region of identity between the paralogs. Figure S7. Breakpoint junction sequencing of second inherited ATAD3B/ATAD3A deletion in Family 2: Alignment to ATAD3B and ATAD3A shows that the breakpoint occurred within a region of identity between the paralogs. Figure S8. Confirmatory array data from Family 3: The heterozygous deletion can be appreciated in the proband and mother’s samples, but not in the father’s sample. Figure S9. Breakpoint junction sequencing of maternally inherited ATAD3B/ATAD3A deletion in Family 3: Alignment to ATAD3B and ATAD3A shows that the breakpoint occurred within a region of identity between the paralogs. Figure S10. Breakpoint junction sequencing of paternally inherited 2-exon deletion in ATAD3A (Family 4): Delineation of the [file 13073_2021_873_MOESM2_ESM.docx]

**Table S2. Primers used for breakpoint junction analyses**

| **Family** | **Forward primer name** | **Forward primer sequence** | **Reverse primer name** | **Reverse primer sequence** |
| --- | --- | --- | --- | --- |
| Family 1 | Family 1_BKPT1_F1 | AGCTCTGCCCTCATCACAGT | Family 1_BKPT1_R1 | AAGTGGGTGCACCTGTCTGT |
| Family 2 and Family 3 | Family 2_BKPT1_F1 | TTGGAGTTCTGTGGTCCTGG | Family 2_BKPT1_R1 | CAGGCCCACACTGCTGAC |
| Family 2 | Family 2_BKPT2_F1 | AAGAATGCGACAGCCGTCA | Family 2_BKPT2_R1 | AGGATGTTCCTGTACAGGCTG |
| Family 4 | Family 4_BKPT1_F1 | GTCCTTGCGTCTGCAGGT | Family 4_BKPT1_R1 | GCCTTTAAAACCTGTGACTGTG |

**Table S3. Missense variants identified in *ATAD3A.*** Bioinformatic predictions are only provided for missense variants, since these have less relevance for indels

| **Position [hg19]** | **Nucleotide*** | **Protein*** | **gnomAD (MAF)** | **gnomAD hom** | **CADD score** | **SIFT** | **MT** | **DANN** | **Revel** | **GERP** |
| --- | --- | --- | --- | --- | --- | --- | --- | --- | --- | --- |
| Chr1:1451415 | c.229C>G | p.(Leu77Val) | 0.0004 | 0 | 22.1 | T | D | D | B | 2.53 |
| Chr1:1447798 | c.150C>G | p.(Phe50Leu) | 0 | 0 | 25.1 | T | D | D | B | 1.95 |
| Chr1:1454364 | c.508C>T | p.(Arg170Trp) | 0.000004 | 0 | 25.5 | D | D | D | B | -0.33 |
| Chr1:1455954 | c.707G>T | p.(Gly236Val) | 0 | 0 | 25.6 | D | D | D | D | 4.42 |
| Chr1:1459235 | c.980G>C | p.(Arg327Pro) | 0 | 0 | 25.8 | D | D | D | D | 4.8 |
| Chr1:1459704 | c.1141dupG | p.(Val381Glyfs*17) | 0 | 0 | - | - | - | - | - | - |
| Chr1:1463151 | c.1414delC | p.(His472fs) | 0.00000398 | 0 | - | - | - | - | - | - |
| Chr1:1469391 | c.1703_1705delAGA | p.(Lys568del) | 0.0000199 | 0 | - | - | - | - | - | - |

* Variant nomenclature provided according to NM_001170535.1.

Abbreviations: B – benign; D – damaging/deleterious; hom – homozygotes; MAF – minor allele frequency; MT – MutationTaster; T – tolerated


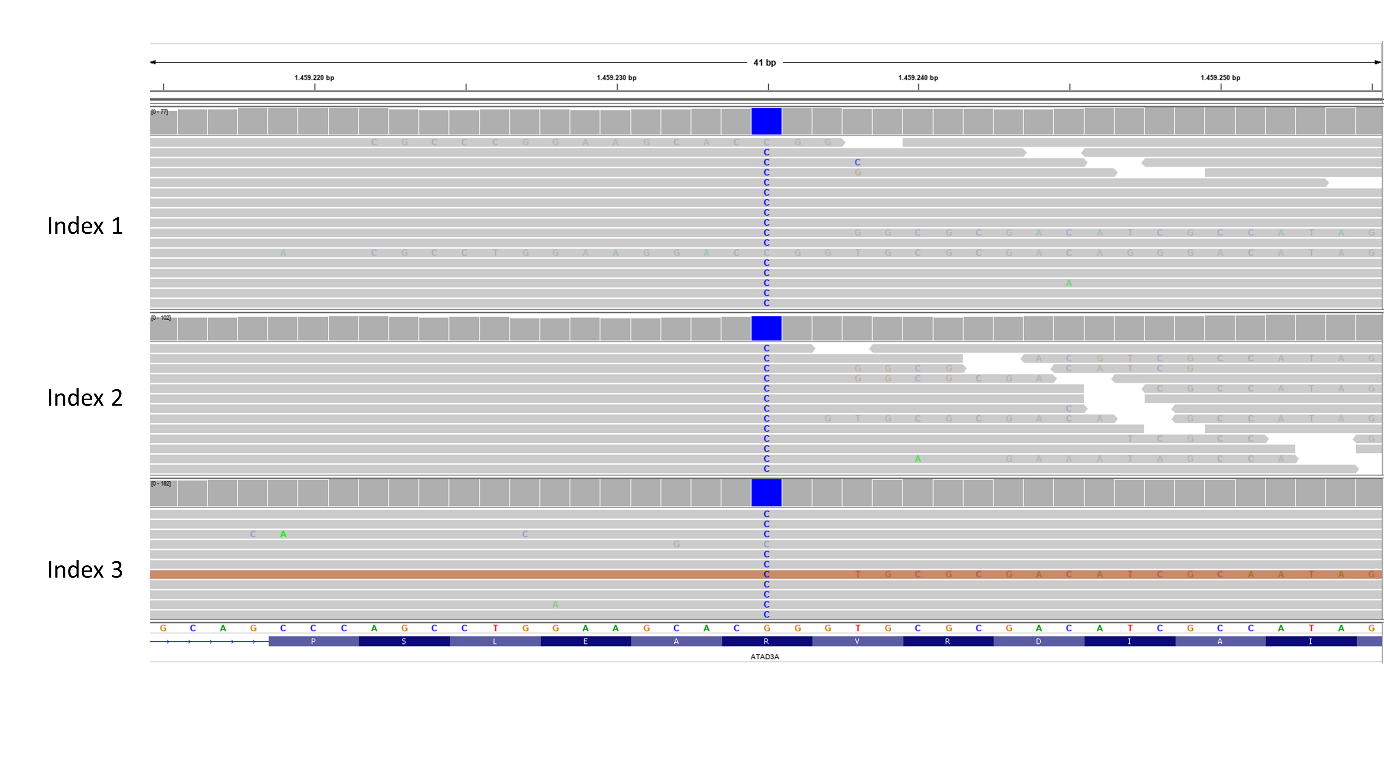


**Figure S1. Homozygous variant in Family 8**. Visualization of exome sequencing reads shows that all three affected individuals form Family 8 are homozygous for the variant c.980G>C, p.(Arg327Pro).


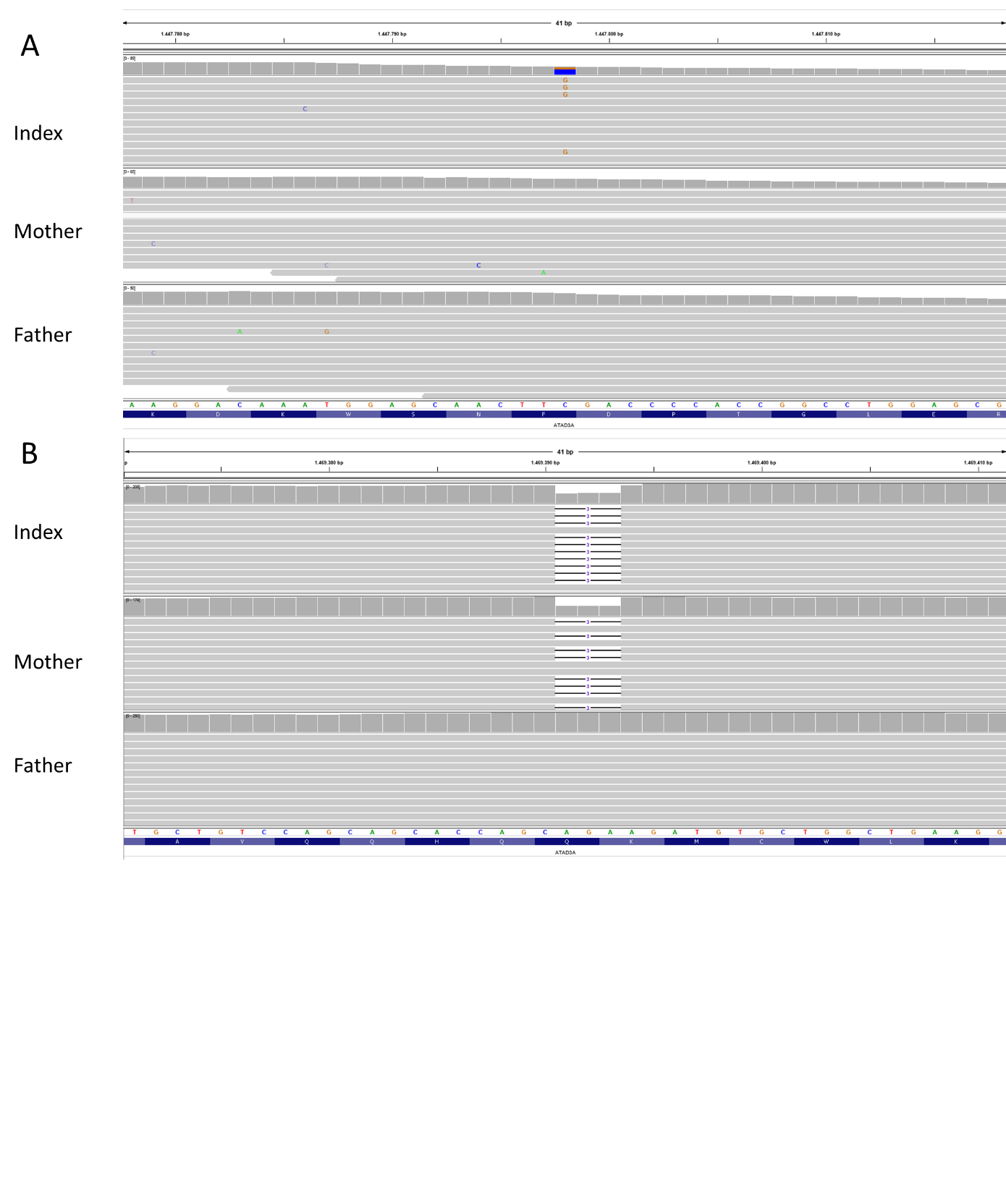


**Figure S2. Segregation analysis in Family 7**. (A) The variant c.150C>G, p.(Phe50Leu), seen in the upper panel (affected proband) could not be detected in DNA from peripheral blood of the parents (middle and lower panels) indicating its *de novo* origin. Total read count was 36, with 11/36 (31%) alternative reads. (B) The variant c.1703_1705del, p.(Lys568del) is maternally inherited. Total read count was 171, with 90/171 (53%) showing alternative allele. Identity by state (IBS) analysis from exome sequencing data showed that the index patient and the father share 52% of alternative alleles, excluding non-paternity.


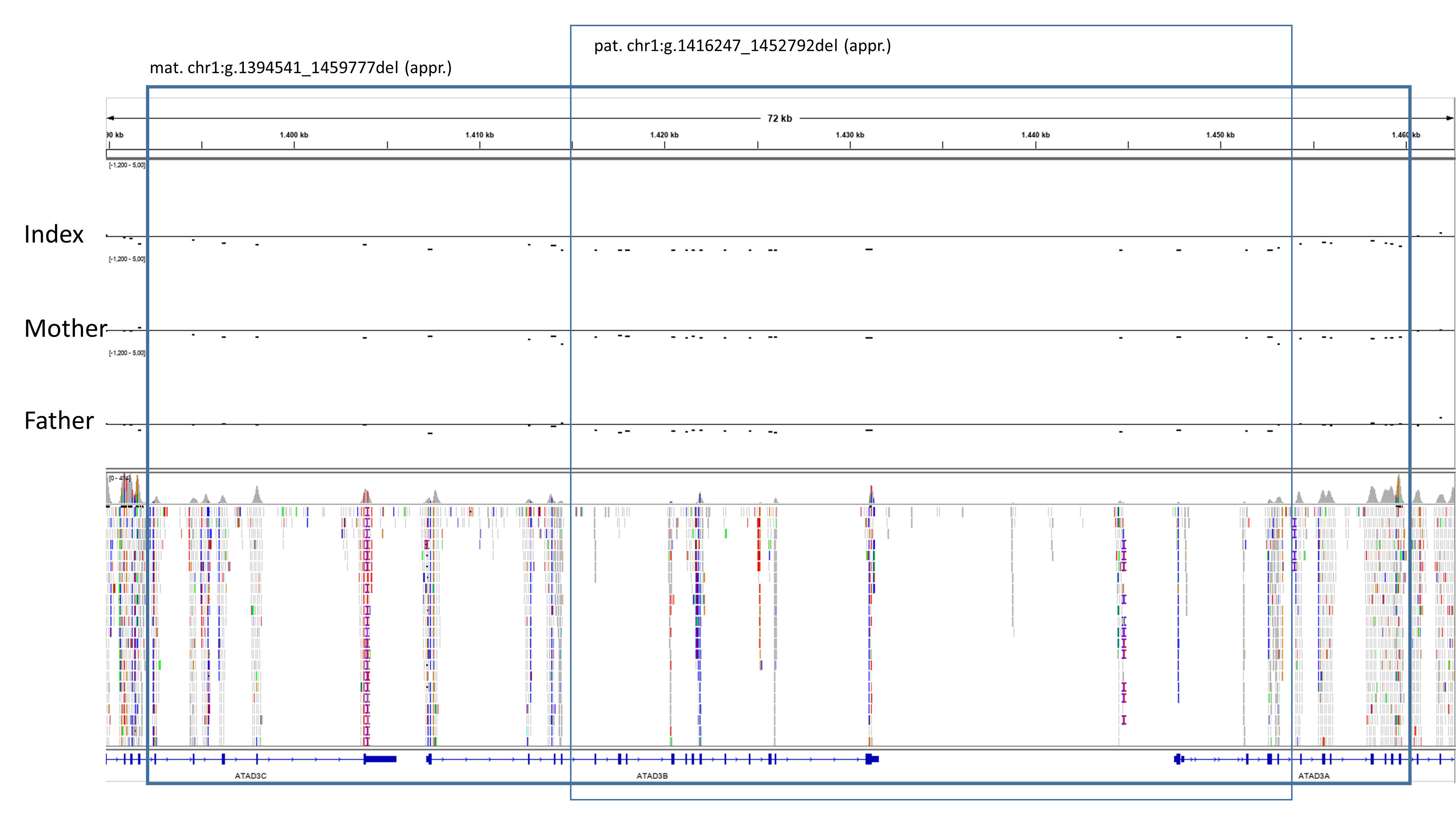


**Figure S3. Compound heterozygous deletion affecting *ATAD3A* in Family 1.** Visualisation of exome sequencing read alignments using the Integrative Genomics Viewer (IGV, bottom panel) at the *ATAD3A*/ *ATAD3B*/ *ATAD3C* gene cluster. CNV calling from exome sequencing data (using ExomeDepth) indicated two overlapping deletions affecting *ATAD3A*: a maternally inherited *ATAD3C*-*ATAD3A* deletion (approximate breakpoint based on IGV and homology between *ATAD3C* and *ATAD3A*: chr1:g.(1391729_1394541)_(1459777_1461841del)), encompassing a smaller, paternally inherited *ATAD3B*-*ATAD3A* deletion (breakpoint junction analysis revealed that NAHR occurred between chr1:1413926-1414584 and chr1:1452593-1453251). These deletions, inherited in trans, are expected to generate two separate fusion genes.

CLUSTAL O(1.2.4) multiple sequence alignment

ATAD3B GCCTTGTGGGGTGGGGTTGGTGTCTGACCTCCCTCCCCGGGGGCCTTCGCAGGCTTCTCT 60

FAMILY1 GCCTTGTGGGGTGGGGTTGGTGTCTGACCTCCCTCCCCGGGGGCCTTCGCAGGCTTCTCT 60

ATAD3A CAGTGCAGTCCAAAAGGGGGTGTCCGGCCTCCCTCCCGGGGGGCCTT--CGCGGGCTTCT 58

* * * ****** * ********** ********* * * ***

ATAD3B GCTGGTGCTTCTGTGCCTGTGGGTCTGGATTCCTCCAGGGCCTGATCCTGGGTGCAGATG 120

FAMILY1 GCTGGTGCTTCTGTGCCTGTGGGTCTGGATTCCTCCAGGGCCTGATCCTGGGTGCAGATG 120

ATAD3A GCTGGTGCTTCTGTGCCTGTGGGTCTGGATTCCTCCAGGGCCTGATCCTGGGTGCAGATG 118

************************************************************

ATAD3B CAGCTGGAAGCCCTGAACCTGCTGCACACACTAGTCTGGGCACGGAGTCTCTGCCGTGCC 180

FAMILY1 CAGCTGGAAGCCCTGAACCTGCTGCACACACTAGTCTGGGCACGGAGTCTCTGCCGTGCC 180

ATAD3A CGGCTGGAAGCCCTGAGCCTGCTGCACACACTAGTCTGGGCATGGAGTCTCTGCCGTGCC 178

* ************** ************************* *****************

ATAD3B GGAGCTGTGCAGACACAGGAGCGGCTGTCAGGCAGTGCCAGCCCTGAGCAAGTGCCAGCT 240

FAMILY1 GGAGCCGTGCAGACACAGGAGCGGCTGTCAGGCAGTGCCAGCCCTGAGCAAGTGCCAGCT 240

ATAD3A GGAGCCGTGCAGACACAGGAGCGGCTGTCAGGCAGTGCCAGCCCTGAGCAAGTGCCAGCT 238

***** ******************************************************

ATAD3B GGTGAGTGCTGTGCTCTGCAGGAGTATGAGGCCGCCGTGGAGCAGCTCAAGAGCGAGCAG 300

FAMILY1 GGTGAGTGCTGTGCTCTGCAGGAGTATGAGGCCGCCGTGGAGCAGCTCAAGAGCGAGCAG 300

ATAD3A GGTGAGTGCTGTGCTCTGCAGGAGTATGAGGCCGCCGTGGAGCAGCTCAAGAGCGAGCAG 298

************************************************************

ATAD3B ATCCGGGCGCAGGCTGAGGAGAGGAGGAAGACCCTGAGCGAGGAGACCCGGCAGCACCAG 360

FAMILY1 ATCCGGGCGCAGGCTGAGGAGAGGAGGAAGACCCTGAGCGAGGAGACCCGGCAGCACCAG 360

ATAD3A ATCCGGGCGCAGGCTGAGGAGAGGAGGAAGACCCTGAGCGAGGAGACCCGGCAGCACCAG 358

************************************************************

ATAD3B GCCGTAAGAGCGCAAGAGGCCGCGAGGGAGGCCGCCCGGCTGCGGGGAGCGGCCTGGGGC 420

FAMILY1 GCCGTAAGAGCGCAAGAGGCCGCGAGGGAGGCCGCCCGGCTGCGGGGAGCGGCCTGGGGC 420

ATAD3A GCCGTAAGAGCGCAAGAGGCCGCGAGGGAGGCCGCCCGGCTGCGGGGAGCGGCCTGGGGC 418

************************************************************

ATAD3B AGGACTGGGAGCTGGGTGTGGTCCCGGGGCACTCTGGAGTCAGCCATTAGAGCTGCCCTC 480

FAMILY1 AGGACTGGGAGCTGGGTGTGGTCCCGGGGCACTCTGGAGTCAGCCATTAGAGCTGCCCTC 480

ATAD3A AGGACTGGGAGCTGGGTGTGGTCCCGGGGCACTCTGGAGTCAGCCATTAGAGCTGCCCTC 478

************************************************************

ATAD3B GGAACGGCCTTGCACAAACGCCTAAGACCTGTAAGGTCCCTCACTGCTGAGCCGGACGGG 540

FAMILY1 GGAACGGCCTTGCACAAACGCCTAAGACCTGTAAGGTCCCTCACTGCTGAGCCGGACGGG 540

ATAD3A GGAACGGCCTTGCACAAACGCCTAAGACCTGTAAGGTCCCTCACTGCTGAGCCGGACGGG 538

************************************************************

ATAD3B AGGTCCCCGCGCCTCCCCACGTTTGTGTGAGGCTGATGGCGCGTCGGAGTCCCCGGCGCT 600

FAMILY1 AGGTCCCCGCGCCTCCCCACGTTTGTGTGAGGCTGATGGCGCGTCGGAGTCCCCGGCGCT 600

ATAD3A AGGTCCCCGCGCCTCCCCACGTTTGTGTGAGGCTGATGGCGCGTCGGAGTCCCCGGCGCT 598

************************************************************

ATAD3B CCGCCCAGTCGGCCCAGACTGCAGCTCCCGGCTGAGATGTGTCTTTGCCGCCCTCTTCTC 660

FAMILY1 CCGCCCAGTCGGCCCAGACTGCAGCTCCCGGCTGAGATGTGTCTTTGCCGCCCTCTTCTC 660

ATAD3A CCGCCCAGTCGGCCCAGACTGCAGCTCCCGGCTGAGATGTGTCTTTGCCGCCCTCTTCTC 658

************************************************************

ATAD3B CCCCAGAGGGCCCAGTATCAAGACAAGCTGGCCCGGCAGCGCTACGAGGACCAACTGAAG 720

FAMILY1 CCCCAGAGGGCCCAGTATCAAGACAAGCTGGCCCGGCAGCGCTACGAGGACCAACTGAAG 720

ATAD3A CCCCAGAGGGCCCAGTATCAAGACAAGCTGGCCCGGCAGCGCTACGAGGACCAACTGAAG 718

************************************************************

ATAD3B CAGCAGGTGAGCTCAGCCTCCCCTGCGAGGCGCCTGCGTCCCTGAGAACGTAGGTGGCTT 780

FAMILY1 CAGCAGGTGAGCTCAGCCTCCCCTGCGAGGCGCCTGCGTCCCTGAGAACGTAGGTGGCTT 780

ATAD3A CAGCAGGTGAGCTCAGCCTCCCCTGCGAGGCGCCTGCGTCCCTGAGAACGTAGGTGGCTT 778

************************************************************

ATAD3B TGTGGGACCAGTCAGTGGGTCAGAGGCCACGGGGCAAGAACGATGGGGTTGCTGACGGTG 840

FAMILY1 TGTGGGACCAGTCAGTGGGTCAGAGGCCACGGGGCAAGAACGCTGGGGTTGCTGACGGTG 840

ATAD3A TGTGGGACCAGTCAGTGGGTCAGAGGCCACGGGGCAAGAACGCTGGGGTTGCTGACGGTG 838

****************************************** *****************

ATAD3B GGTGCTAGAGCAGGGGAAACTACTCGGACAGACACGCACCAGCACACGTGTACAGGCACA 900

FAMILY1 GGTGCTAGAGCAGGGGAAACTACTCGGACAGACACGCACCAGCACACGTGTACAGGCACA 900

ATAD3A GGTGCTAGAGCAGGGGAAACTACTCGGACAGACACGCACCAGCACACGTGTACAGGCACA 898

************************************************************

ATAD3B CATGCAGATGTGTGCACACATGTACACGGAGACACAGGCACCTGCCCACACAGACACACA 960

FAMILY1 CATGCAGACGTGTGCACACATGTACATGGAGACACAGGCACCTGCCCACACGGACACACA 960

ATAD3A CATGCAGACGTGTGCACACATGTACATGGAGACACAGGCACCTACCCACACGGACACACA 958

******** ***************** **************** ******* ********

ATAD3B CTCCTCGCACACACACTCCCGGCAGACAGGCACACACACCCCTGCA

FAMILY1 CTCCTCGCACACACACTCCCAGCACACACAGACAGGTGCACCCACT

ATAD3A CTCCTCGCACACACACTCCCAGCACACACAGACAGGTGCACCCACT

******************** *** *** *** *

**Figure S4. Breakpoint junction sequencing of paternally inherited *ATAD3B/ATAD3A* deletion in Family 1.** Blue font indicates alignment to *ATAD3B,* red font indicates alignment to *ATAD3A.* Breakpoint occurred within the region of identity corresponding to chr1:1413926-1414584 and chr1:1452593-1453251.


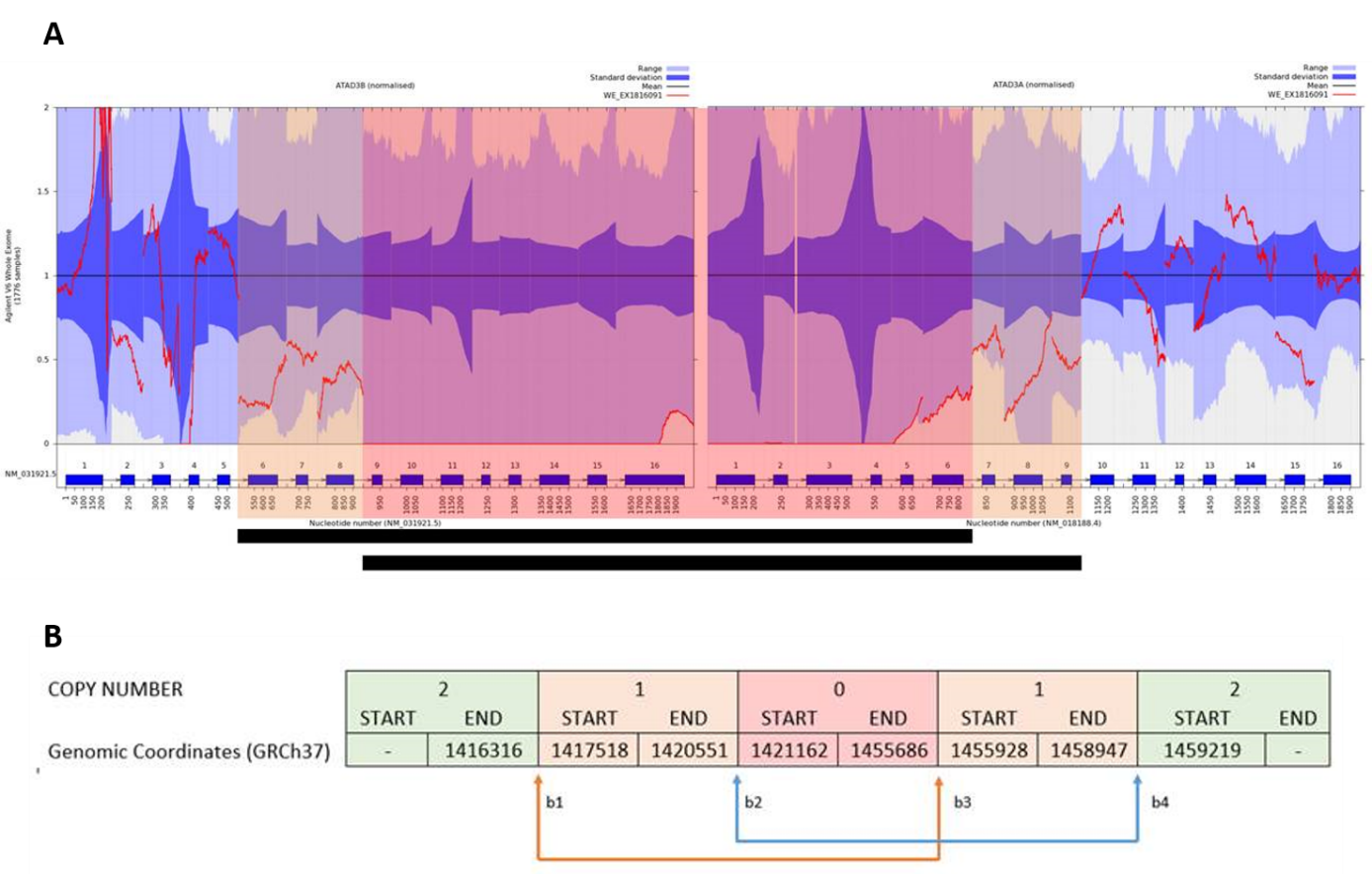


**Figure S5. Read depth analysis of exome sequencing data in Family 2.** (A) Read depth analysis suggested a compound heterozygous deletion in Family 2, resulting in a biallelic deletion of the first exons of *ATAD3A.* (B) Assumed breakpoint junctions as assigned from exome data (before breakpoint junction analysis). CNV calling in the region is sub-optimal due to the high homology between the paralogs. Sanger sequencing of the breakpoint junctions revealed accurate breakpoint coordinates (see figures below). The first breakpoint occurred within the region of identity corresponding to chr1:1416206-1416369 and chr1:1454260-1454423. The second breakpoint occurred within the region of identity corresponding to chr1:1420558-1420661 and chr1:1458286-1458389.

CLUSTAL O(1.2.4) multiple sequence alignment

ATAD3B GGTGTTGAGCATTTTTCTGGTTTTAAAGGCTTTTCTCTTTTTCTGCGGCTTCTTCTCAGC 60

FAMILY2 GGTGTTGAGCATTTTTCTGGTTTTAAAGGCTTTTCTCTTTTTCTGCGGCTTCTTCTCAGC 60

ATAD3A CACATGGGCACAGTCACAGGTTTTAAAGGCTTTTCTCTTTTTCTGCGGCTTCTTCTCAGC 60

* * * * ******************************************

ATAD3B AACTTCTCAATGAGGAGAATTTACGGAAGCAGGAGGAGTCCGTGCAGAAGCAGGAAGCCA 120

FAMILY2 AACTTCTCAATGAGGAGAATTTACGGAAGCAGGAGGAGTCCGTGCAGAAGCAGGAAGCCA 120

ATAD3A AACTTCTCAATGAGGAGAATTTACGGAAGCAGGAGGAGTCCGTGCAGAAGCAGGAAGCCA 120

************************************************************

ATAD3B TGCGGCGAGGTAGGCTGTCTGCTCTCCTGGCTGGGGCGGAGGTGGCGGGGGCTGCTTGTG 180

FAMILY2 TGCGGCGAGGTAGGCTGTCTGCTCTCCTGGCTGGGGCGGAGGTGGCGGGGGCTGCTTGTG 180

ATAD3A TGCGGCGAGGTAGGCTGTCTGCTCTCCTGGCTGGGGCGGAGGTGGCGGGGGCTGCTTGTG 180

************************************************************

ATAD3B GATCCGGCGTGCACTCTGAGCCTGAGTTCTGCCGCCCGGCCCCTCATAGCTACCAGTGCA 240

FAMILY2 GACCCGGCGTGCACTCTGAGCCTGAGTTCTGCCGCCCGGCCCCTCATAGCTACCAGTGCA 240

ATAD3A GACCCGGCGTGCACTCTGAGCCTGAGTTCTGCCGCCCGGCCCCTCATAGCTACCAGTGCA 240

** *********************************************************

ATAD3B GTGGGCGAGGCCTGCTGGGGCTCTGCGGGGTGGGGCTCCCTCTCGGAAGACACCTCTGTC 300

FAMILY2 GTGGGCGAGGCCTGCTGGGGCTCCGCGGGGTGGGGCTGCCTCTCGGAAGACACCTCTGTC 300

ATAD3A GTGGGCGAGGCCTGCTGGGGCTCCGCGGGGTGGGGCTGCCTCTCGGAAGACACCTCTGTC 300

*********************** ************* **********************

ATAD3B TGCGAGTGGACGCCAGGATCTGTTCAGGGAGGGCAGGAGCTGCTTCACTTCATGGGAAGT 360

FAMILY2 TGCGAGTGGACGCCAGGATCTGTTCAGGGAGGGCAGGAGCTGCTTCACTTCATGGGAAGT 360

ATAD3A TGCGAGTGGACGCCAGGATCTGTTCAGGGAGGGCAGGAGCTGCTTCACTTCATGGGAAGT 360

************************************************************

ATAD3B ACAGGGGCCTTTTTTTTTTTTTTGAGACGGAGTCTCGCTCTGTCACCCAGGCAGGAGTGC 420

FAMILY2 ACAGGGGCCTTTTTTTTTTTTTTGAGACGGAGTCTCGCTCTGTCACCCAGGCAGGAGTGC 420

ATAD3A ACAGGGGCCTTTTTTTTTTTTTTGAGACGGAGTCTCGCTCTGTCACCCAGGCAGGAGTGC 420

************************************************************

ATAD3B AATAGCACGATCTCAGCTCACTGCAACCTCTGCCTCCCAGGTTTAAGCAATTCTCCTGCC 480

FAMILY2 AATAGCACGATCTCAGCTCACTGCAACCTCTGCCTCCCAGGTTTAAGCAATTCTCCTGCC 480

ATAD3A AATAGCACGATCTCAGCTCACTGCAACCTCTGCCTCCCAGGTTTAAGCAATTCTCCTGCC 480

************************************************************

ATAD3B TCAGCCTCCCGAGTAGCTGGGATTATAGGCTCCCGCCACCACGCCCAGCTAATTTTTTTG 540

FAMILY2 TCAGCCTCCCGAGTAGCTGGGATTATAGGCTCCCGCCACCACGCCCAGCTAATTTTTTTG 540

ATAD3A TCAGCCTCCCGAGTAGCTGGGATTATAGGCTCCCGCCACCACGCCCAGCTAATTTTTTTG 540

************************************************************

ATAD3B TATCTTCAGTAGAGAAAGGGTTTCACTGTGTTGGCCAGGCTGGTCTTGAACTTCTTGATC 600

FAMILY2 TATCTTCAGTAGAGAAAGGGTTTCACTGTGTTGGCCAGGCTGGTCTTGAACTTCTTGATC 600

ATAD3A TATCTTCAGTAGAGAAAGGGTTTCACTGTGTTGGCCAGGCTGGTCTTGAACTTCTTGATC 600

************************************************************

ATAD3B TCATTATCCGCCTGCCTTGGCCTTCCACAGTGCTGGGATTACAGGCGTGAGCCTCTGCGT 660

FAMILY2 TCATTATCCGCCTGCCTTGGCCTTCCACAGTGCTGGGATTACAGGCGTGAGCCTCTGCGT 660

ATAD3A TCATTATCCGCCTGCCTTGGCCTTCCACAGTGCTGGGATTACAGGCGTGAGCCTCTGCGT 660

************************************************************

ATAD3B TCTGCCTAGAACATGGGTCTTTACTGTCCTGGTTTCAGTGGGGATCACAGGTATTTGGTG 720

FAMILY2 TCTGCCTAGAACATGGGTCTTTACTGTCCTGGTTTCAGTGGGGATCACAGGTATTTGGTG 720

ATAD3A TCTGCCTAGAACATGGGTCTTTACTGTCCTGGTTTCAGTGGGGATCACAGGTATTTGGTG 720

************************************************************

ATAD3B CCATGTGGCATTTGTTGGCGAGTGCTCCAGGCAAACGTCTGTCACCACTCTTCACCGTGG 780

FAMILY2 CCATGTGGCATTTGTTGGCGAGTGCTCCAGGCAAACGTCTGTCACCACTCTTCACCGTGG 780

ATAD3A CCATGTGGCATTTGTTGGCGAGTGCTCCAGGCAAACGTCTGTCACCACTCTTCACCGTGG 780

************************************************************

ATAD3B GTGGGCTTGTGGCGAGGTGTGTGCGTTTAATGTTCAGTAGCCAGGCACGTGGCACGTCAC 840

FAMILY2 GTGGGCTTGTGGCGAGGTGTGTGCGTTTAATGTTCAGTAGCCAGGCACGTGGCACGTCAC 840

ATAD3A GTGGGCTTGTGGCGAGGTGTGTGCGTTTAATGTTCAGTAGCCAGGCACGTGGCACGTCAC 840

************************************************************

ATAD3B GCGTGTCTGAGTTCTGACAGCTGTGTTTCTGTGTGAGGGGGGCTTCCTTCAGAACTCCGC 900

FAMILY2 GCGTGTCTGAGTTCTGACAGCTGTGTTTCTGTGTGAGGGGGGCTTCCTTCAGAACTCCGC 900

ATAD3A GCGTGTCTGAGTTCTGACAGCTGTGTTTCTGTGTGAGGGGGGCTTCCTTCAGAACTCCGC 900

************************************************************

ATAD3B GTTCTGGTTTTTTGCTTCAAAGAGCTCGTCCTGAGAAGTTGCCTAGGCCTCTGGGTCGGA 960

FAMILY2 GTTCTGGTTTTTTGCTTCAAAGAGCTCGTCCTGAGAAGTTGCCTAGGCCTCTGGGTCGGA 960

ATAD3A GTTCTGGTTTTTTGCTTCAAAGAGCTCGTCCTGAGAAGTTGCCTAGGCCTCTGGGTCGGA 960

************************************************************

ATAD3B TTTCTGCCCTAATCCATGGGCAGGGCCGGCCTGTGGCGCTGTCCCTACCAAGGTCTGTGT 1020

FAMILY2 TTTCTGCCCTAATCCATGGGCAGGGCCGGCCTGTGGCGCTGTCCCTACCAAGGTCTGTGT 1020

ATAD3A TTTCTGCCCTAATCCATGGGCAGGGCCGGCCTGTGGCGCTGTCCCTACCAAGGTCTGTGT 1020

************************************************************

ATAD3B GTGTCTGTGGCACGGGCCTGTCCATGGACTGGGCTTGTCCGTGGAGTGGGTCG 1073

FAMILY2 GTGTCTGTGGCATGGACCTGTCCGTGGCCTTAGCCTATTGGCGGCGTGGGCCT 1073

ATAD3A GTGTCTGTGGCATGGACCTGTCCGTGGCCTTAGCCTATTGGCGGCGTGGGCCT 1073

************ ** ******* *** ** ** * * * ** ***** *

**Figure S6. Breakpoint junction sequencing of first *ATAD3B/ATAD3A* deletion in Family 2.** Blue font indicates alignment to *ATAD3B,* red font indicates alignment to *ATAD3A.* Breakpoint occurred within the region of identity corresponding to chr1:1416206-1416369 and chr1:1454260-1454423.

CLUSTAL O(1.2.4) multiple sequence alignment

ATAD3B AGTCTTGTTTTCCAGGAATAAAGTACcATTTTTAGTGGCCAAGGATGTACCAGAGGGTGT 60

FAMILY2 AGTCTTGTTTTCCAGGAATAAAGTACCATTTTTAGTGGCCAAGGATGTACCAGAGGGTGT 60

ATAD3A AGTCTTGTTTTCCAGGAATAAAGTACcATTTTTAGTGGCCAAGaATGTACCAGAGGGTGT 60

******************************************* ****************

ATAD3B GGCCCTGTGACATCCAGCTGGGTCTGCCCAGGGCCCCGCTCAGCGACCGAGGCTTTCTAG 120

FAMILY2 GGCCCTGTGACATCCAGCTGGGTCTGCCCAGGGCCCCGCTCAGCGACCGAGGCTTTCTAG 120

ATAD3A GGCCCTGTGACATCCgGCTGGGTCTGtCCAGGGCCCCGCTCAGCGACCGAGGCTTTCTAG 120

*************** ********** *********************************

ATAD3B GATTTATGCTGCCAGTTGCAgAGAAAATGGCCCtGAGTGAGGGCGTTATGACTGCCCCAC 180

FAMILY2 GATTTATGCTGCCAGTTGCAAAGAAAATGGCCCAGAGTGAGGGCGTTATGACTGCCCCAC 180

ATAD3A GATTTATGCTGCCAGTTGCAgAGAAAATGGCCCtGAGTGAGGGCGcTgTGACTGCCCCAC 180

******************** ************ *********** * ************

ATAD3B CTGCCTCCTGTAACCGCGTGGCTGTGGGATTCGGGGCTGGGAATTCGGGTTCCTGTGGGG 240

FAMILY2 CTGCCTCCTGTAACCGCGTGGCTGTGGGATTCGGGGCTGGGAATTCGGGTTCCTGTGGGG 240

ATAD3A CTGCCTCCTGTAACCGCGTGGCTGTGGGATTCGGGGCcGGGAATTCGcGTTCCTGTGGGG 240

************************************* ********* ************

ATAD3B CCAGCACACGGCCCTGTGCTTCTCCCTCAGGCGGAGAGAGGGTGGGGGCAGCCCCGTGCG 300

FAMILY2 CCAGCACACGGCCCTGTGCTTCTCCCTCAGGCGGAGAGAGGGTGGGGGCAGCCCCGTGCG 300

ATAD3A CCAGtgCACGGCCCTGTGCTTCTCCCTCgGGCGGAGAGAGGGTGGGGGCAGCCCCGTGCG 300

**** ********************** *******************************

ATAD3B TCTCCTGCTCTAGGAGGGAGGGACGGTGGGGGCCGGTGCGCCAGTGCGGTGTCTCTGCTG 360

FAMILY2 TCTCCTGCTCTAGGAGGGAGGGACGGTGGGGGCCGGTGCGCCAGTGCGGTGTCTCTGCTG 360

ATAD3A TCTCCTGCTCTAaGAGGGAGGGACGGTGGGGGCCGGTGCGCCAGTGCGGTGTCTCTGCTG 360

************ ***********************************************

ATAD3B CAGGTGGCTGGGCTGACGCTGCTGGCTGTCGGGGTCTACTCAGCCAAGAATGCGACAGCC 420

FAMILY2 CAGGTGGCTGGGCTGACGCTGCTGGCTGTCGGGGTCTACTCAGCCAAGAATGCGACAGCC 420

ATAD3A CAGGTGGCTGGGCTGACGCTGCTGGCTGTtGGGGTCTACTCAGCCAAGAATGCcACgctt 420

***************************** *********************** **

ATAD3B GTCACTGGCCGCTTCATCGAGGCTCGGCTGGGGAAGCCGTCCCTAGTGAGGGAGACGTCC 480

FAMILY2 GTCACTGGCCGCTTCATCGAGGCTCGGCTGGGGAAGCCGTCCCTAGTGAGGGAGACGTCC 480

ATAD3A GTCgCcGGCCGCTTCATCGAGGCTCGGCTGGGGAAGCCGTCCCTAGTGAGGGAGACGTCC 480

*** * ******************************************************

ATAD3B CGCATCACGGTGCTGGAGGCGCTGCGGCACCCCATCCAGGTAGCGGCGCAGGCCTGGCCC 540

FAMILY2 CGCATCACGGTGCTGGAGGCGCTGCGGCACCCCATCCAGGTAGCGGCGCAGGCCTGGCCC 540

ATAD3A CGCATCACGGTGCTtGAGGCGCTGCGGCACCCCATCCAGGTAGCaGCGCAGGCCTGGCCC 540

************** ***************************** ***************

ATAD3B TCCCTGAGTGCAGTTCCTGGCTGAGTCCCTTCTGCCCCACGAGCACAGCCCACGCACACC 600

FAMILY2 TCCCTGAGTGCAGTTCCTGGCTGAGTCCCTTCTGCCCCACGAGCACAGCCCACGCACACC 600

ATAD3A TCCCTGAGTGCAGTTCCTGGCTGAGTCCCTTCTGCCCCACGAGCACAGCCCACGCACACC 600

************************************************************

ATAD3B CTCCCGTCCCTTCCCTTTCCCCGGATAACaGGCACCCGCACgCTGCTTCACGGGTGGGTT 660

FAMILY2 CTCCCGTCCCTTCCCTTTCCCCGGATAACGGGCACCCGCACACTGCTTCACGGGTGGGTT 660

ATAD3A CTCCCGTCCCTTCCCTTTCCCCGGATAACGGGCACCCGCACACTGCTTCACGGGTGGGTT 660

***************************** *********** ******************

ATAD3B TTCCTGTCTGGCGCTGTACCTTAGGGGTCTGCATCAGTGAGACCCTTCCCCTGTCTGCCT 720

FAMILY2 TTCCTGTCTGGCGCTGTACCTTAGGGGTCTGCATCAGTGAGACCCTTCCCCTGTCTGCCT 720

ATAD3A TTCCTGTCTGGCGCTGTACCTTAGGGGTCTGCATCAGTGAGACCCTTCCCCTGTCTGCCT 720

************************************************************

ATAD3B CGGTGTCCCTTGCTCAGGGCTCTTGATGGGGCCTGGGAGCACATCGGGGTCCTTGCAAGA 780

FAMILY2 CGGTGTCCCCTGCTCAGGGCTCTTGATGGGGCCTGGGAGCACATCGGGGTCCTTGCAAGA 780

ATAD3A CGGTGTCCCCTGCTCAGGGCTCTTGATGGGGCCTGGGAGCACATCGGGGTCCTTGCAAGA 780

********* **************************************************

ATAD3B CCCGGGACTTGGGTGTGCGGCCGTCTGTCAGGGAGCTGCTACAGGCCATGGCGTCTGGT 840

FAMILY2 CCCGGGACTTGGGTGTGCGGCCGTCTATCAGGAAAGCTGCTACAGGCCACGGCGTCTGGT 840

ATAD3A CCCGGGACTTGGGTGTGCGGCCGTCTATCAGGgAAGCTGCTACAGGCCACGGCGTCTGGT 840

************************** ** ** **************** **********

**Figure S7. Breakpoint junction sequencing of second inherited *ATAD3B/ATAD3A* deletion in Family 2.** Blue font indicates alignment to *ATAD3B,* red font indicates alignment to *ATAD3A.* Breakpoint occurred within the region of identity corresponding to chr1:1420558-1420661 and chr1:1458286-1458389.


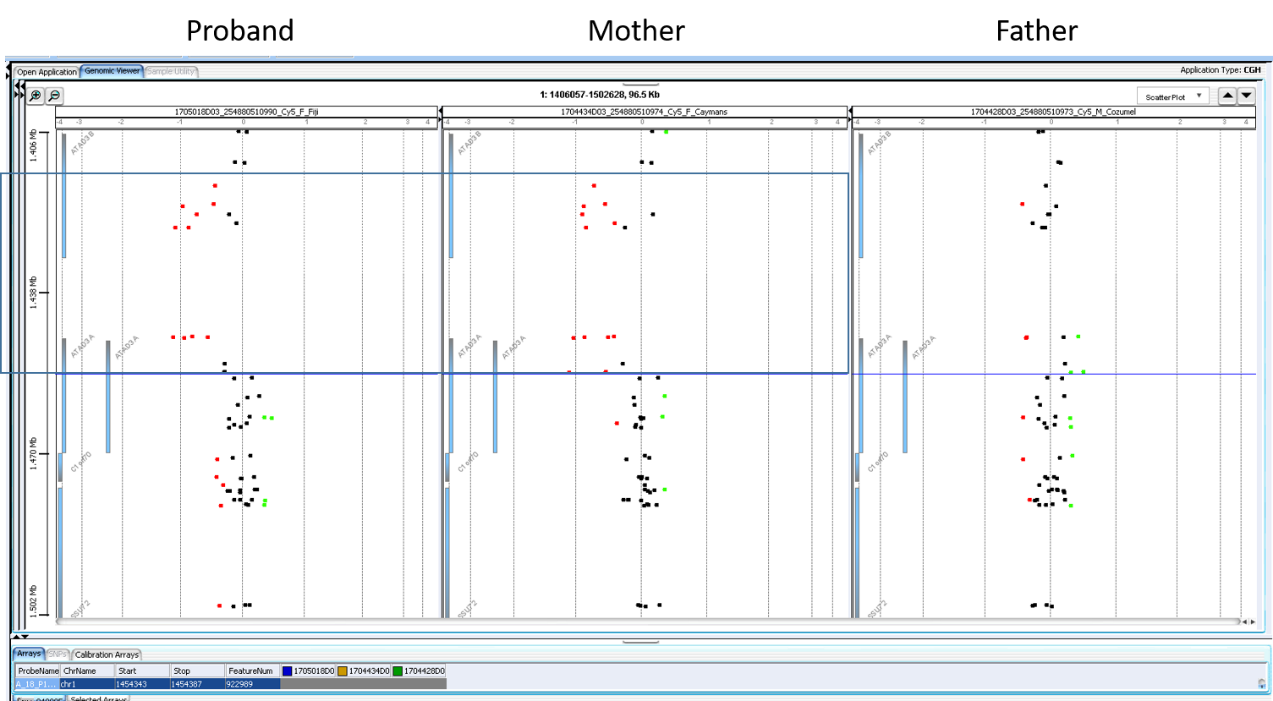


**Figure S8. Confirmatory array data from Family 3.** Heterozygous deletion can be appreciated in proband and mother’s samples. The father does not have a deletion in this region. Array data is suboptimal due to the segmental duplications.

CLUSTAL O(1.2.4) multiple sequence alignment

ATAD3B GTTGAGCATTTTTCTGGTTTTAAAGGCTTTTCTCTTTTTCTGCGGCTTCTTCTCAGCAAC 60

Family3 GTTGAGCATTTTTCTGGTTTTAAAGGCTTTTCTCTTTTTCTGCGGCTTCTTCTCAGCAAC 60

ATAD3A ATGGGCACAGTCACAGGTTTTAAAGGCTTTTCTCTTTTTCTGCGGCTTCTTCTCAGCAAC 60

* * * * *********************************************

ATAD3B TTCTCAATGAGGAGAATTTACGGAAGCAGGAGGAGTCCGTGCAGAAGCAGGAAGCCATGC 120

Family3 TTCTCAATGAGGAGAATTTACGGAAGCAGGAGGAGTCCGTGCAGAAGCAGGAAGCCATGC 120

ATAD3A TTCTCAATGAGGAGAATTTACGGAAGCAGGAGGAGTCCGTGCAGAAGCAGGAAGCCATGC 120

************************************************************

ATAD3B GGCGAGGTAGGCTGTCTGCTCTCCTGGCTGGGGCGGAGGTGGCGGGGGCTGCTTGTGGAT 180

Family3 GGCGAGGTAGGCTGTCTGCTCTCCTGGCTGGGGCGGAGGTGGCGGGGGCTGCTTGTGGAC 180

ATAD3A GGCGAGGTAGGCTGTCTGCTCTCCTGGCTGGGGCGGAGGTGGCGGGGGCTGCTTGTGGAC 180

***********************************************************

ATAD3B CCGGCGTGCACTCTGAGCCTGAGTTCTGCCGCCCGGCCCCTCATAGCTACCAGTGCAGTG 240

Family3 CCGGCGTGCACTCTGAGCCTGAGTTCTGCCGCCCGGCCCCTCATAGCTACCAGTGCAGTG 240

ATAD3A CCGGCGTGCACTCTGAGCCTGAGTTCTGCCGCCCGGCCCCTCATAGCTACCAGTGCAGTG 240

************************************************************

ATAD3B GGCGAGGCCTGCTGGGGCTCTGCGGGGTGGGGCTCCCTCTCGGAAGACACCTCTGTCTGC 300

Family3 GGCGAGGCCTGCTGGGGCTCCGCGGGGTGGGGCTGCCTCTCGGAAGACACCTCTGTCTGC 300

ATAD3A GGCGAGGCCTGCTGGGGCTCCGCGGGGTGGGGCTGCCTCTCGGAAGACACCTCTGTCTGC 300

******************** ************* *************************

ATAD3B GAGTGGACGCCAGGATCTGTTCAGGGAGGGCAGGAGCTGCTTCACTTCATGGGAAGTACA 360

Family3 GAGTGGACGCCAGGATCTGTTCAGGGAGGGCAGGAGCTGCTTCACTTCATGGGAAGTACA 360

ATAD3A GAGTGGACGCCAGGATCTGTTCAGGGAGGGCAGGAGCTGCTTCACTTCATGGGAAGTACA 360

************************************************************

ATAD3B GGGGCCTTTTTTTTTTTTTTGAGACGGAGTCTCGCTCTGTCACCCAGGCAGGAGTGCAAT 420

Family3 GGGGCCTTTTTTTTTTTTTTGAGACGGAGTCTCGCTCTGTCACCCAGGCAGGAGTGCAAT 420

ATAD3A GGGGCCTTTTTTTTTTTTTTGAGACGGAGTCTCGCTCTGTCACCCAGGCAGGAGTGCAAT 420

************************************************************

ATAD3B AGCACGATCTCAGCTCACTGCAACCTCTGCCTCCCAGGTTTAAGCAATTCTCCTGCCTCA 480

Family3 AGCACGATCTCAGCTCACTGCAACCTCTGCCTCCCAGGTTTAAGCAATTCTCCTGCCTCA 480

ATAD3A AGCACGATCTCAGCTCACTGCAACCTCTGCCTCCCAGGTTTAAGCAATTCTCCTGCCTCA 480

************************************************************

ATAD3B GCCTCCCGAGTAGCTGGGATTATAGGCTCCCGCCACCACGCCCAGCTAATTTTTTTGTAT 540

Family3 GCCTCCCGAGTAGCTGGGATTATAGGCTCCCGCCACCACGCCCAGCTAATTTTTTTGTAT 540

ATAD3A GCCTCCCGAGTAGCTGGGATTATAGGCTCCCGCCACCACGCCCAGCTAATTTTTTTGTAT 540

************************************************************

ATAD3B CTTCAGTAGAGAAAGGGTTTCACTGTGTTGGCCAGGCTGGTCTTGAACTTCTTGATCTCA 600

Family3 CTTCAGTAGAGAAAGGGTTTCACTGTGTTGGCCAGGCTGGTCTTGAACTTCTTGATCTCA 600

ATAD3A CTTCAGTAGAGAAAGGGTTTCACTGTGTTGGCCAGGCTGGTCTTGAACTTCTTGATCTCA 600

************************************************************

ATAD3B TTATCCGCCTGCCTTGGCCTTCCACAGTGCTGGGATTACAGGCGTGAGCCTCTGCGTTCT 660

Family3 TTATCCGCCTGCCTTGGCCTTCCACAGTGCTGGGATTACAGGCGTGAGCCTCTGCGTTCT 660

ATAD3A TTATCCGCCTGCCTTGGCCTTCCACAGTGCTGGGATTACAGGCGTGAGCCTCTGCGTTCT 660

************************************************************

ATAD3B GCCTAGAACATGGGTCTTTACTGTCCTGGTTTCAGTGGGGATCACAGGTATTTGGTGCCA 720

Family3 GCCTAGAACATGGGTCTTTACTGTCCTGGTTTCAGTGGGGATCACAGGTATTTGGTGCCA 720

ATAD3A GCCTAGAACATGGGTCTTTACTGTCCTGGTTTCAGTGGGGATCACAGGTATTTGGTGCCA 720

************************************************************

ATAD3B TGTGGCATTTGTTGGCGAGTGCTCCAGGCAAACGTCTGTCACCACTCTTCACCGTGGGTG 780

Family3 TGTGGCATTTGTTGGCGAGTGCTCCAGGCAAACGTCTGTCACCACTCTTCACCGTGGGTG 780

ATAD3A TGTGGCATTTGTTGGCGAGTGCTCCAGGCAAACGTCTGTCACCACTCTTCACCGTGGGTG 780

************************************************************

ATAD3B GGCTTGTGGCGAGGTGTGTGCGTTTAATGTTCAGTAGCCAGGCACGTGGCACGTCACGCG 840

Family3 GGCTTGTGGCGAGGTGTGTGCGTTTAATGTTCAGTAGCCAGGCACGTGGCACGTCACGCG 840

ATAD3A GGCTTGTGGCGAGGTGTGTGCGTTTAATGTTCAGTAGCCAGGCACGTGGCACGTCACGCG 840

************************************************************

ATAD3B TGTCTGAGTTCTGACAGCTGTGTTTCTGTGTGAGGGGGGCTTCCTTCAGAACTCCGCGTT 900

Family3 TGTCTCAGTTCTGACAGCTGTGTTTCTGTGTGAGGGGGGCTTCCTTCAGAACTCCGCGTT 900

ATAD3A TGTCTGAGTTCTGACAGCTGTGTTTCTGTGTGAGGGGGGCTTCCTTCAGAACTCCGCGTT 900

***** ******************************************************

ATAD3B CTGGTTTTTTGCTTCAAAGAGCTCGTCCTGAGAAGTTGCCTAGGCCTCTGGGTCGGATTT 960

Family3 CTGGTTTTTTGCTTCAAAGAGCTCGTCCTGAGAAGTTGCCTAGGCCTCTGGGTCGGATTT 960

ATAD3A CTGGTTTTTTGCTTCAAAGAGCTCGTCCTGAGAAGTTGCCTAGGCCTCTGGGTCGGATTT 960

************************************************************

ATAD3B CTGCCCTAATCCATGGGCAGGGCCGGCCTGTGGCGCTGTCCCTACCAAGGTCTGTGTGTG 1020

Family3 CTGCCCTAATCCATGGGCAGGGCCGGCCTGTGGCGCTGTCCCTACCAAGGTCTGTGTGTG 1020

ATAD3A CTGCCCTAATCCATGGGCAGGGCCGGCCTGTGGCGCTGTCCCTACCAAGGTCTGTGTGTG 1020

************************************************************

ATAD3B TCTGTGGCACGGGCCTGTCCATGGACTGGG

Family3 TCTGTGGCATGGACCTGTCCGTGGCCTTAG

ATAD3A TCTGTGGCATGGACCTGTCCGTGGCCTTAG

********* ** ******* *** ** *

**Figure S9. Breakpoint junction sequencing of maternally inherited *ATAD3B/ATAD3A* deletion in Family 3.** Blue font indicates alignment to *ATAD3B,* red font indicates alignment to *ATAD3A.* Breakpoint occurred within the region of identity corresponding to chr1:1416206-1416369 and chr1:1454260-1454423.

**
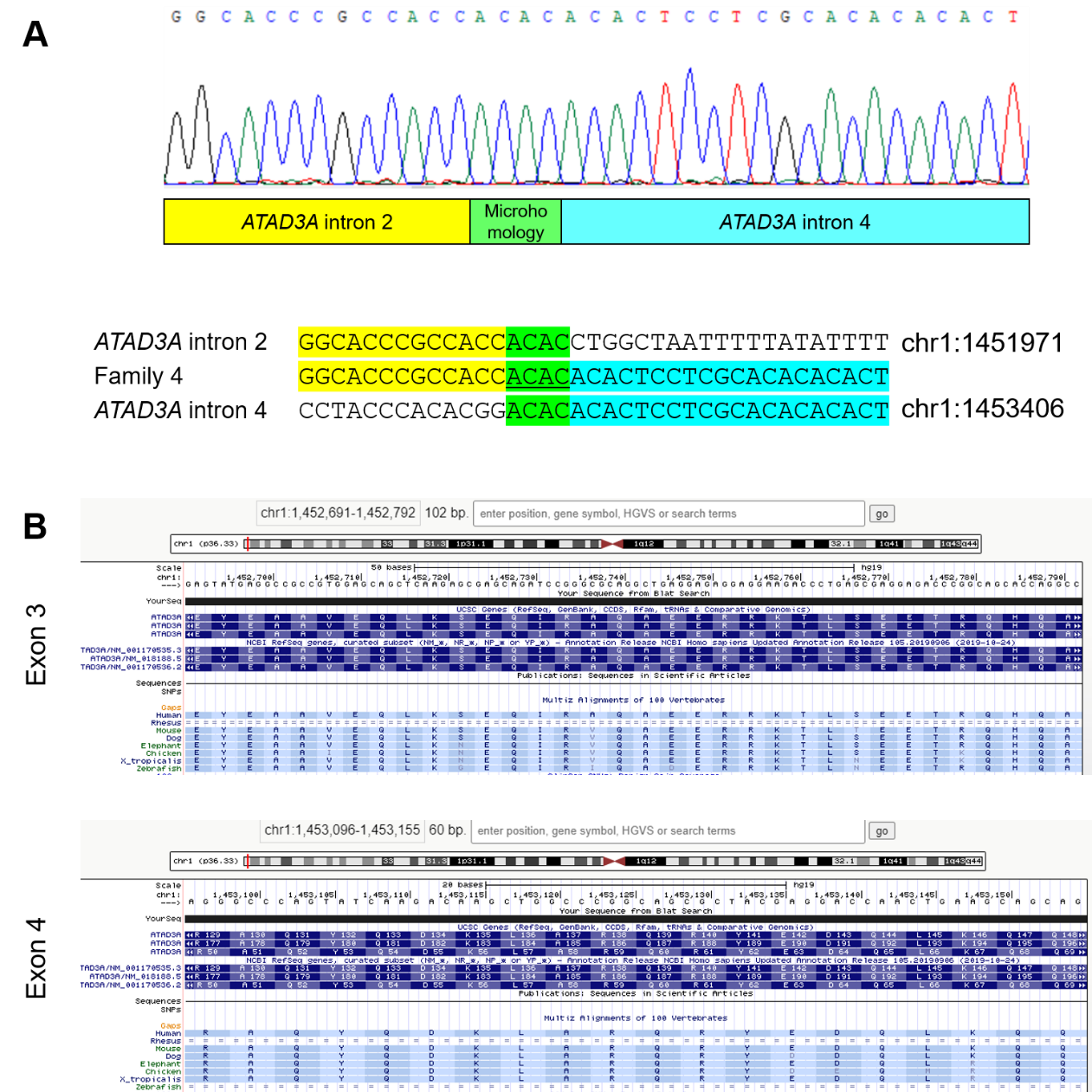
**

**Figure S10. Breakpoint junction sequencing of paternally inherited 2-exon deletion in *ATAD3A* (Family 4).** (A) Sanger trace of DNA breakpoint junction in Family 4, indicating skipping of exons 3 and 4. Yellow highlight shows alignment to *ATAD3A* intron 2*,* blue highlight shows alignment to *ATAD3A* intron 4*.* The mutational signature includes microhomology of four nucleotides (green), implicating fork stalling and template switching (FoSTeS) or microhomology-mediated break-induced replication (MMBIR) as the underlying mutational mechanism. (B) Evolutionary conservation of exons 3 and 4, which are skipped as a result of the deletion.


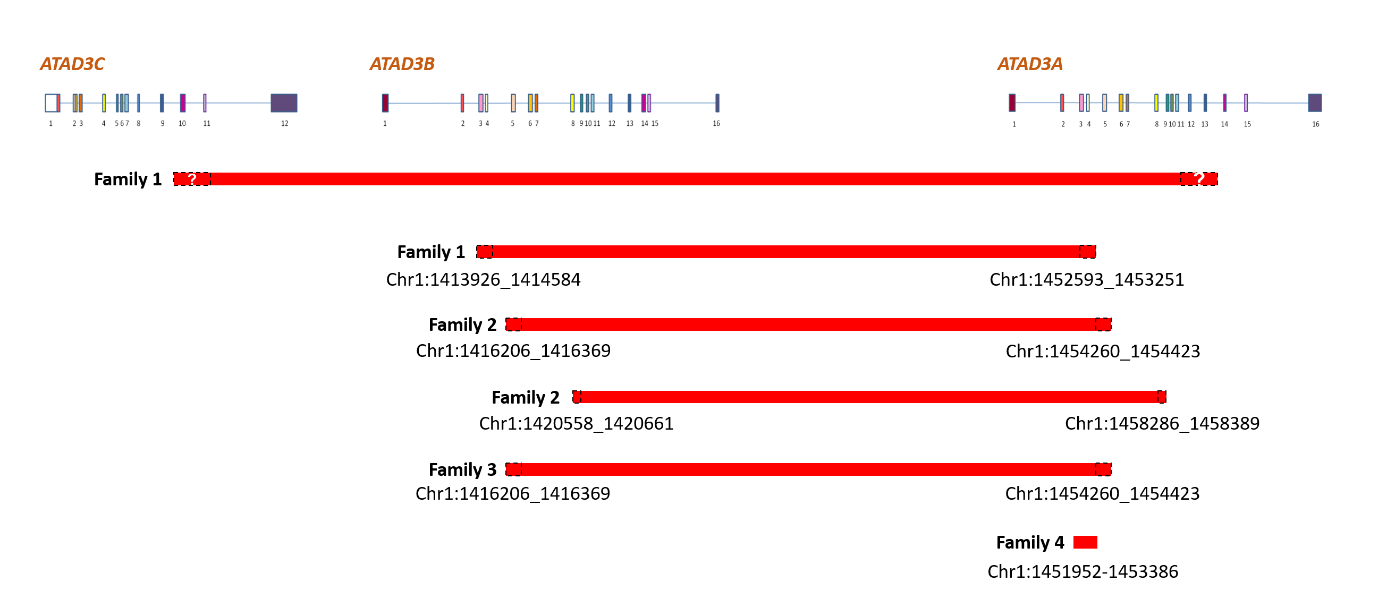


**Figure S11. Schematic diagram of all CNVs identified in this study.** 5 of the 6 CNVs were resolved at the breakpoint junction level.
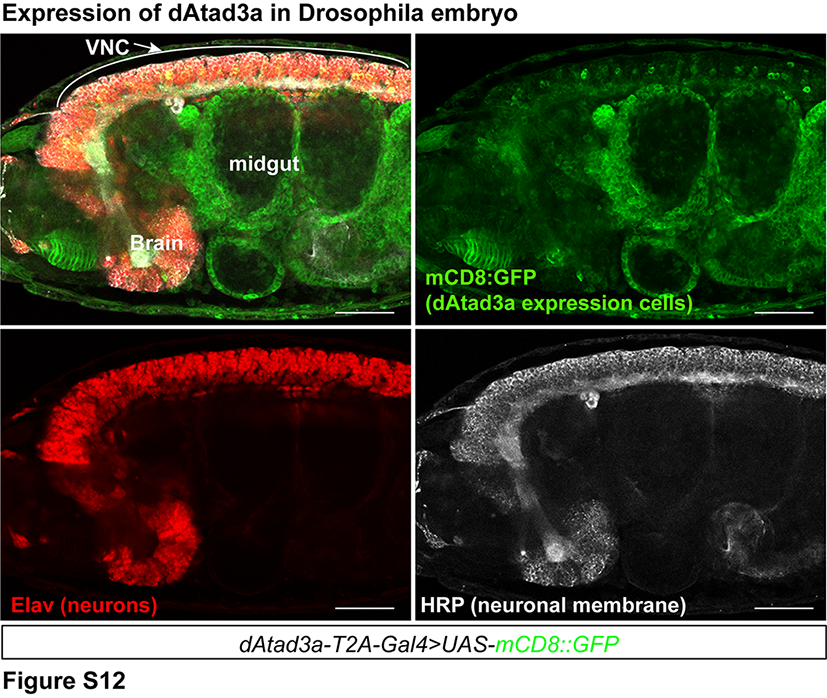


**Figure S12. dAtad3a is expressed ubiquitously in embryos**

Confocal micrographs of an embryo expressing GFP protein (green) under the control of *dAtad3a-T2A-Gal4 (dAtad3a-T2A-Gal4/UAS-mCD8::GFP*). Elav (red) stained neurons. HRP stained neuronal membranes. VNC indicates ventral nerve cord. Scale bars indicate 50 μm.

**
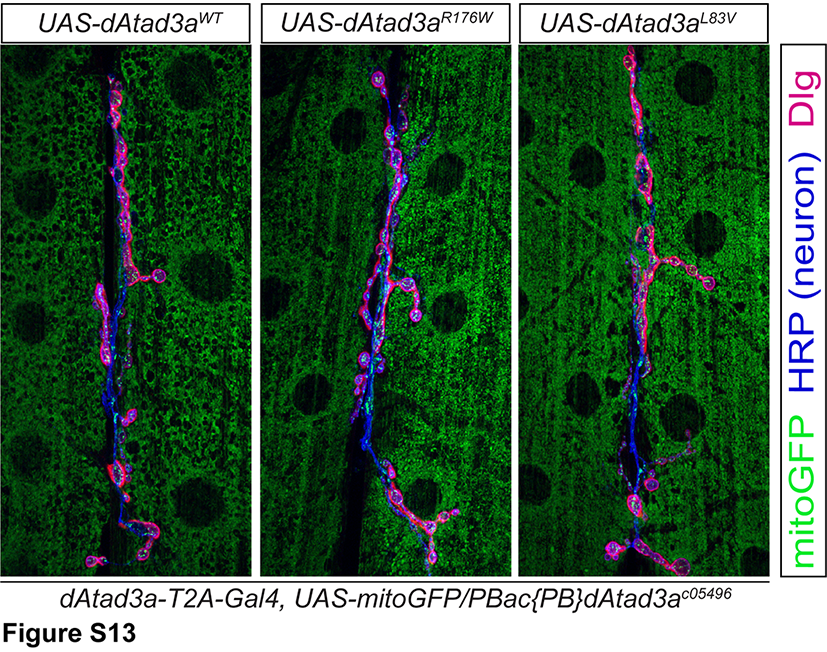
**

**Figure S13. R176W and L83V did not affect mitochondria content and morphology in larvae muscles**

Confocal micrographs of *dAtad3a* mutant larvae muscles expressing *dAtad3a^WT^*, *dAtad3a^R176W^*, or *dAtad3a^L83V^*. mitoGFP (green) labels mitochondria. Dlg (red) labels boutons. HRP (blue) labels neurons.

**
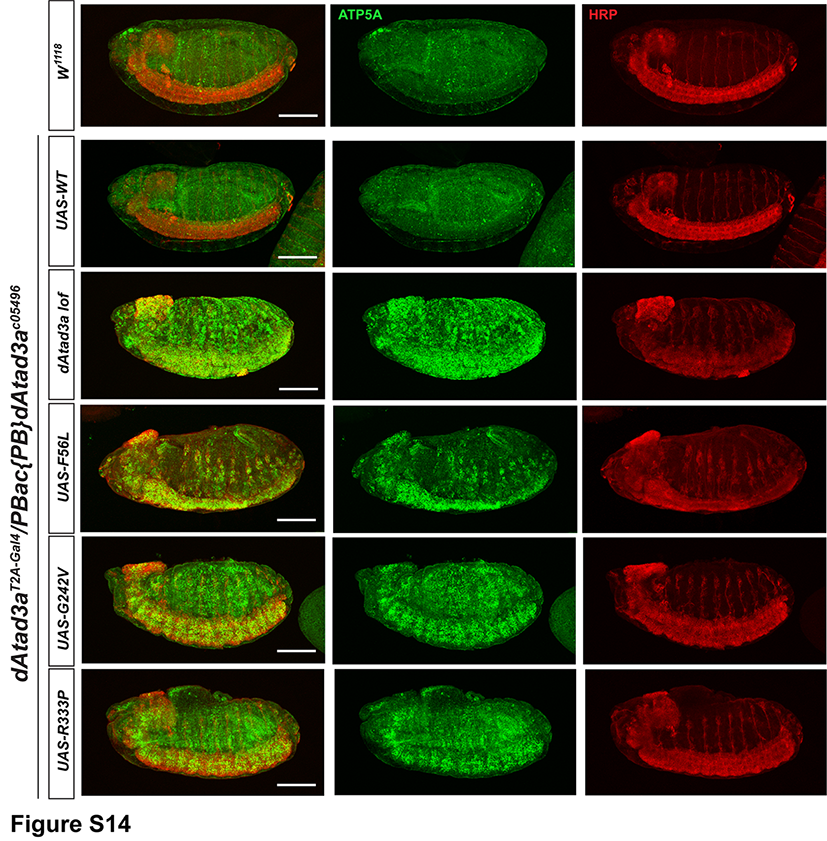
**

**Figure S14. dAtad3a null, F56L, G242V, and R333P cause increased mitochondrial content in embryos**

Confocal micrographs of *w^1118^*, *dAtad3a* null mutant embryos and those expressing *dAtad3a^WT^*, *dAtad3a^F56L^*, *dAtad3a^G242V^*, or *dAtad3a^R333P^*. ATP5A (green) stained mitochondria, and anti-HRP (red) stained neuronal membranes. Scale bars indicate 100 μm.

**
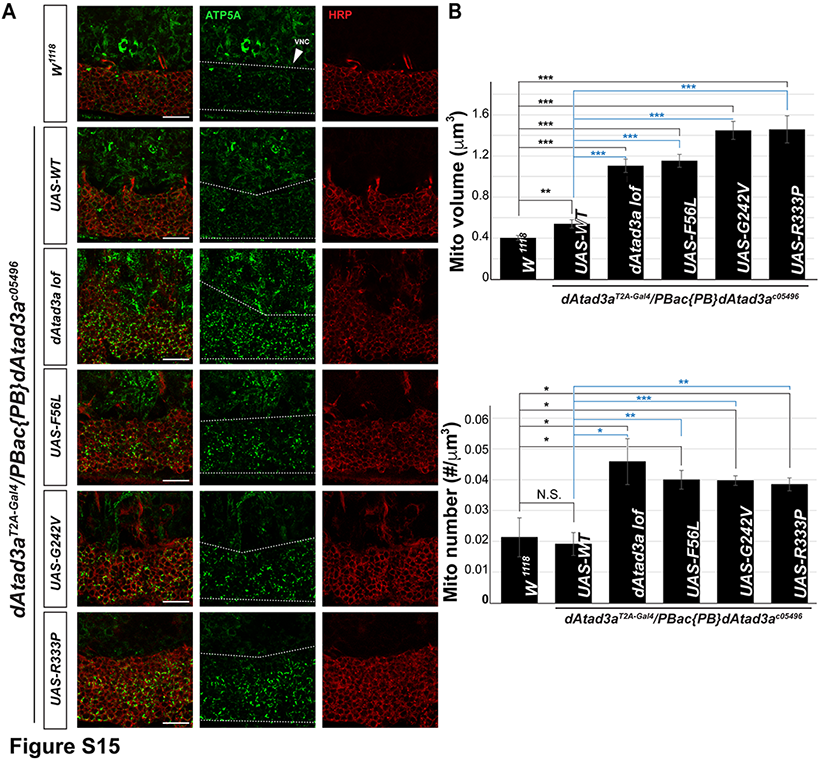
**

**Figure S15. dAtad3a null, F56L, G242V, and R333P cause increased mitochondrial numbers and size in embryos**

(A) Magnified VNC region of *w^1118^*, *dAtad3a* null mutant embryos and those expressing *dAtad3a^WT^*, *dAtad3a^F56L^*, *dAtad3a^G242V^*, or *dAtad3a^R333P^*. The arrow and white lines indicate VNC. Scale bars indicate 20 μm. (B) Quantification of mitochondrial volume and number. Error bars indicate SEM. P values were calculated using Student’s t-test. **P* <0.05, ***P* <0.01, ****P* <0.001. Numbers of mitochondria for the analyses are as followed: *w^1118^* (n=916), WT (n=960), null (n=1318), F56L (n=1435), G242V (n=1426), and R333P (n=1657).

**
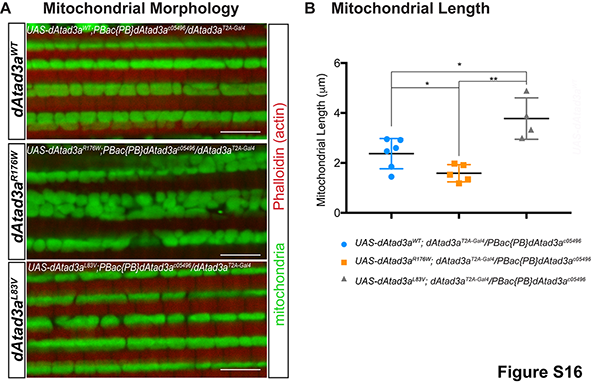
**

**Figure S16. R176W causes small mitochondria in adult muscles**

(A) Confocal micrographs of thorax muscle from 5-day-old flies - *dAtad3a* mutants expressing *dAtad3a^WT^*, *dAtad3a^R176W^*, or *dAtad3a^L83V^*. mitoGFP (green) labels mitochondria. Phalloidin (red) labels actin. Scale bar, 10 μm. (B) Quantification of mitochondrial length for *dAtad3a* mutant adult expressing *dAtad3a^WT^*, *dAtad3a^R176W^*, or *dAtad3a^L83V^*. Error bars indicate SEM. P values were calculated using Student’s t-test. **P* <0.05, ***P* <0.01, ****P* <0.001.

**
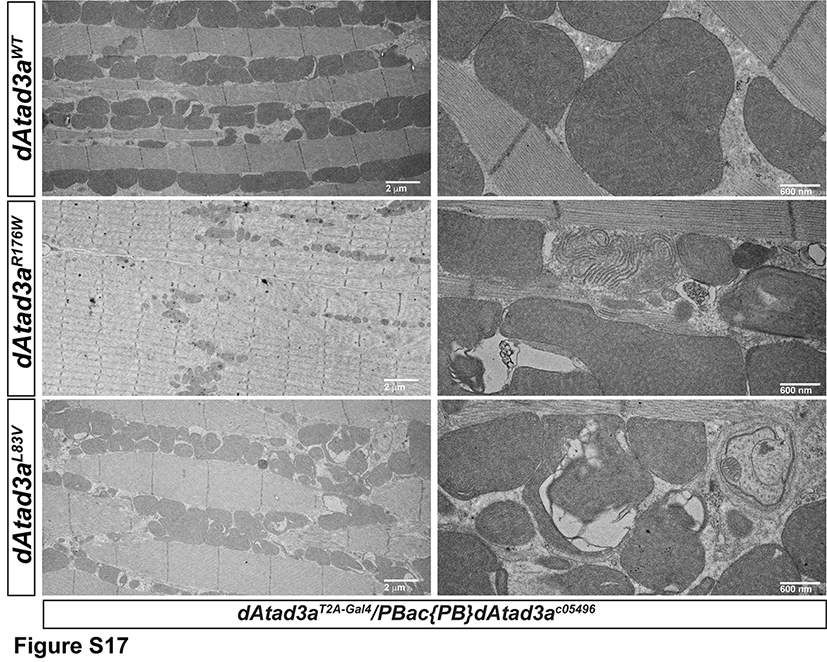
**

**Figure S17. R176W and L83V cause various defects in mitochondria in adult muscles**

Electron micrographs of thorax muscles from 8 week old *dAtad3a* mutant flies expressing *dAtad3a^WT^*, *dAtad3a^R176W^*, or dAtad3a^L83V^. Scale bar, 2μm (left), and 600 nm (right).
